# Oral Irrigation Devices: A Scoping Review

**DOI:** 10.1002/cre2.912

**Published:** 2024-06-16

**Authors:** Farzana Sarkisova, Zac Morse, Kevin Lee, Nagihan Bostanci

**Affiliations:** ^1^ Department of Oral Health Auckland University of Technology Auckland New Zealand; ^2^ Department of Food Science and Microbiology Auckland University of Technology Auckland New Zealand; ^3^ Department of Dental Medicine, Division of Oral Health and Periodontology Karolinska Institutet Stockholm Sweden

**Keywords:** dental devices, home care, dental plaque, oral hygiene, periodontitis

## Abstract

**Objectives:**

Self‐performed oral hygiene is essential for preventing dental caries, periodontal, and peri‐implant diseases. Oral irrigators are adjunctive oral home care aids that may benefit oral health. However, the effects of oral irrigation on oral health, its role in oral home care, and its mechanism of action are not fully understood. A comprehensive search of the literature revealed no existing broad scoping reviews on oral irrigators. Therefore, this study aimed to provide a comprehensive systematic review of the literature on oral irrigation devices and identify evidence gaps.

**Methods:**

The Joanna Briggs Institute and Preferred Reporting Items for Systematic reviews and Meta‐Analyses extension for Scoping Reviews guidelines were utilized to prepare the review. Four databases and eight gray literature sources were searched for English publications across any geographical location or setting.

**Results:**

Two hundred and seventy‐five sources were included, predominantly from scientific journals and academic settings. Most studies originated from North America. Research primarily involved adults, with limited studies in children and adolescents. Oral irrigation was safe and well‐accepted when used appropriately. It reduced periodontal inflammation, potentially by modulating the oral microbiota, but further research needs to clarify its mechanism of action. Promising results were reported in populations with dental implants and special needs. Patient acceptance appeared high, but standardized patient‐reported outcome measures were rarely used. Anti‐inflammatory benefits occurred consistently across populations and irrigant solutions. Plaque reduction findings were mixed, potentially reflecting differences in study designs and devices.

**Conclusions:**

Oral irrigators reduce periodontal inflammation, but their impact on plaque removal remains unclear. Well‐designed, sufficiently powered trials of appropriate duration need to assess the clinical, microbiological, and inflammatory responses of the periodontium to oral irrigation, particularly those with periodontitis, dental implants, and special needs. Patient‐reported outcome measures, costs, caries prevention, and environmental impact of oral irrigation need to be compared to other oral hygiene aids.

## 1 Introduction

Despite being largely preventable, dental caries, gingivitis, and periodontitis remain the most prevalent chronic noncommunicable diseases worldwide (Bernabe et al. 2020). Not only can these diseases lead to edentulism, masticatory dysfunction, and disability, but they can also place a significant financial burden on individuals and the global economy. For example, global yearly costs of dental diseases reached a staggering USD544.41 billion in 2015 (Righolt et al. 2018). Furthermore, oral diseases negatively affect systemic health (Manoil et al. 2021; Silbereisen et al. 2023).

Dental biofilms are complex multispecies microbial communities forming on nonshedding tooth surfaces (Belibasakis et al. 2023). Dysbiotic dental biofilm is involved in the pathogenesis of both caries and periodontitis, with regular biofilm disruption playing a crucial role in preventing these diseases. As emphasized in the European Federation of Periodontology (EFP) S3 level clinical practice guideline for the treatment of Stage I–III periodontitis, adequate self‐performed oral hygiene is essential for optimal response to periodontal treatment and secondary prevention of periodontitis, as individuals successfully treated for periodontitis remain at an increased risk for the disease recurrence (Sanz et al. 2020). As periodontitis affects approximately half of people worldwide and negatively impacts oral and systemic health (Petersen and Ogawa 2012; Tonetti et al. 2017), effective and well‐accepted oral home care measures are essential for this population. However, due to the scarcity of high‐quality evidence, the EFP guideline does not make a strong conclusion on any specific interdental oral hygiene aid for individuals in periodontal maintenance (Sanz et al. 2020). Similarly, a Cochrane review on the effectiveness of interdental cleaning aids for caries and periodontal diseases prevention published in 2019 could not identify any studies assessing caries, with authors calling for long‐term trials to assess this outcome (Worthington et al. 2019).

Another common group of diseases induced by biofilms are peri‐implant diseases, with the prevalence of peri‐implant mucositis being 46.83% and peri‐implantitis 19.83% (Lee et al. 2017). The diseases may be associated with considerable morbidity and treatment costs (Herrera et al. 2023). Furthermore, peri‐implantitis can progress significantly faster than periodontitis, and its progression will often lead to implant loss (Schwarz et al. 2018). The EFP S3 level clinical practice guideline for the prevention and treatment of peri‐implant diseases emphasizes that appropriate biofilm control is essential for preserving peri‐implant health and achieving optimal peri‐implant treatment outcomes (Herrera et al. 2023). However, despite the importance of self‐performed oral care in this population, the guideline states it is not known which method of home care is the most effective in individuals treated for peri‐implantitis due to the absence of studies comparing different home care methods.

Toothbrushing can effectively disrupt biofilms and, thus, prevent the development or arrest of the progression of dental caries and periodontal diseases (Worthington et al. 2019). However, a toothbrush cannot disrupt biofilm on interproximal surfaces of the teeth and prostheses in close contact and may not access some hard‐to‐reach areas. This presents a significant problem, with approximal surfaces commonly affected by dental caries and periodontitis (Lorentz et al. 2009; Pretty and Ekstrand 2016). Adjunctive tools, such as interdental brushes and string floss, should supplement toothbrushing in these areas (Worthington et al. 2019). However, these tools require a certain level of dexterity from the operator and may be challenging to use in hard‐to‐reach areas. Furthermore, all oral hygiene aids have the potential to cause trauma, which necessitates monitoring their use and tailoring oral home care to the patient's situation (Sanz et al. 2020).

In addition, certain populations may experience difficulties maintaining good oral hygiene due to low manual dexterity or factors hindering appropriate biofilm control. For example, fixed orthodontic appliances increase oral disease risk by retaining dental biofilm (Mulimani and Popowics 2022). Meticulous oral hygiene is required in this population to prevent dental caries and periodontal diseases, but it may be challenging to achieve. Another example is intermaxillary fixation, which makes acceptable oral hygiene nearly impossible (Tsolov 2022). Further, individuals with mental and physical disabilities, who collectively represent about 16% of the global population (World Health Organization 2023), may not be able to maintain acceptable oral hygiene with conventional oral hygiene aids (Buda 2016). Additionally, both EFP guidelines for the prevention and treatment of peri‐implant diseases and treatment of Stage I–III periodontitis emphasize that clinicians need to consider their client's personal preferences when recommending oral home care aids to support compliance and adherence to oral hygiene measures (Herrera et al. 2023; Sanz et al. 2020). Therefore, oral health practitioners need to have a wide variety of aids to choose from.

One of the adjunctive aids, which is considered to be generally safe, well‐accepted by patients, and able to access hard‐to‐reach areas, is an oral irrigation device (Herrera et al. 2023; Sanz et al. 2020). The US Food & Drug Administration (2023) defines it as an electric device that creates “a pressurised stream of water to remove food particles between the teeth and promote good periodontal condition.” Oral irrigators (OIs) were launched into the market in 1962 (Ciancio 2009). They perform cleaning by a pulsating or continuous stream of pressured water and can also deliver antimicrobial solutions subgingivally. Most self‐contained oral irrigation units available in the market today are pulsating devices. The pulsating action of these devices produces a compression and decompression phase, which is key to their efficacy (Lyle 2012; Mancinelli‐Lyle et al. 2023).

Regular oral irrigation may reduce periodontal and peri‐implant inflammation (Gennai et al. 2023; Slot, Valkenburg, and Van der Weijden 2020). Adjunctive irrigation is suggested for individuals with periodontitis and peri‐implant mucositis when interdental brushes cannot be used or in addition to other home care measures (Herrera et al. 2023; Sanz et al. 2020). However, evidence regarding these devices' biofilm removal ability and mechanism of action has been inconclusive, with experts calling for more clinical studies on OIs (Gennai et al. 2023; Herrera et al. 2023; Sanz et al. 2020; Slot, Valkenburg, and Van der Weijden 2020).

To avoid wasting resources and prioritize areas requiring further investigation, future research should be guided by the available evidence and existing knowledge gaps (Feres et al. 2022). However, it is unclear what kind of research has been conducted on OIs, as no broad systematic reviews could be identified on the topic. Some existing reviews covered multiple oral hygiene aids and did not focus on a detailed investigation of OIs (Gennai et al. 2023; Slot, Valkenburg, and Van der Weijden 2020; Van der Weijden and van Loveren 2023). Reviews that have focused exclusively on OIs either answered narrow questions concerning OIs' efficacy and safety or focused on a specific condition or population and, therefore, included a limited number of studies identified through restricted search strategies (Allen 2019; AlMoharib et al. 2023; Husseini, Slot, and Van der Weijden 2008; Jolkovsky and Lyle 2015). A comprehensive scoping review is needed to map the evidence that has accumulated on these devices since their launch in the market and to identify research gaps to guide future research. Therefore, this scoping review aims to systematically map the evidence on oral irrigation devices and identify any research gaps.

## 2 Methods

The scoping review was conducted following the Joanna Briggs Institute (JBI) guidance for scoping reviews (Aromataris and Munn 2020), and the manuscript was prepared using the PRISMA‐ScR (Preferred Reporting Items for Systematic reviews and Meta‐Analyses extension for Scoping Reviews) guidelines (Tricco et al. 2018).

The research question was established using the JBI's ‘population, concept, context’ framework (Aromataris and Munn 2020). The concept identified was “oral irrigation devices.” Population and context are irrelevant as this review aims to identify a broad range of evidence on OIs irrespective of the population and setting. This review aims to answer the following primary research question: “What is known about oral irrigation devices?”; the subquestions addressed are: “What methodologies have been used in research? What research gaps exist concerning oral irrigation devices? What aspects require further investigation?” Primary and secondary research published in English across any geographical location or setting was considered for inclusion; letters, narratives, opinion pieces, commentaries, guidelines, registered trials with no results available, and historical reviews were excluded.

The search strategy was developed with the assistance of a health sciences librarian. The main searches of four databases (CINAHL, the Dentistry and Oral Sciences Source, MEDLINE, and Scopus) and eight sources of gray literature (Cochrane Library, Cochrane Oral Health's Trials Register, Google, Google Scholar, the ISRCTN Registry, Open Gray, ProQuest Dissertations and Theses Global, and the WHO Clinical Trials Registry Platform) were conducted on January 12, 2022, with no date limiters applied, with a follow‐up search to capture newly published sources on December 23 and 24, 2023. In addition, citation searching for eligible sources was performed via Scopus. The search strategy is presented in Table 1.

**Table 1:** Search strategy.

Search number	Search terms
1	“Water irrig*” OR “Water floss*” OR (“therapeutic irrig*” AND water) OR “oral irrigat*” OR monojet OR “subgingival irrigat*” OR “subgingival tip” OR “dental irrigat*” OR “interdental device*” OR “gingiv* therapeutic irrigation” OR “interdental cleaning device*” OR (“interdental cleaning” AND water) OR (“interdental cleaning” AND irrigat*) OR (“inter‐dental cleaning” AND water) OR (“inter‐dental cleaning” AND irrigat*) OR “irrigation device*” OR “supragingival irrigat*” OR “pulsated jet” OR “microdroplet device*” OR “micro droplet device*” OR “Water pik” OR waterpik OR waterpick OR “water pick” OR “water jet” OR waterjet OR “perio pik” OR “pick pocket” OR pickpocket OR “pik pocket” OR pikpocket
2	“Oral health” OR dental OR dentistry OR caries OR periodont* OR gingiv* OR “oral hygiene”

*Note:* Search terms used for CINAHL, the Dentistry and Oral Sciences Source, MEDLINE, and Scopus; Steps 1 and 2 were combined for the final search. The gray literature search was conducted using the same search terms, with the search strategy customized for each database.

**Figure 1:**
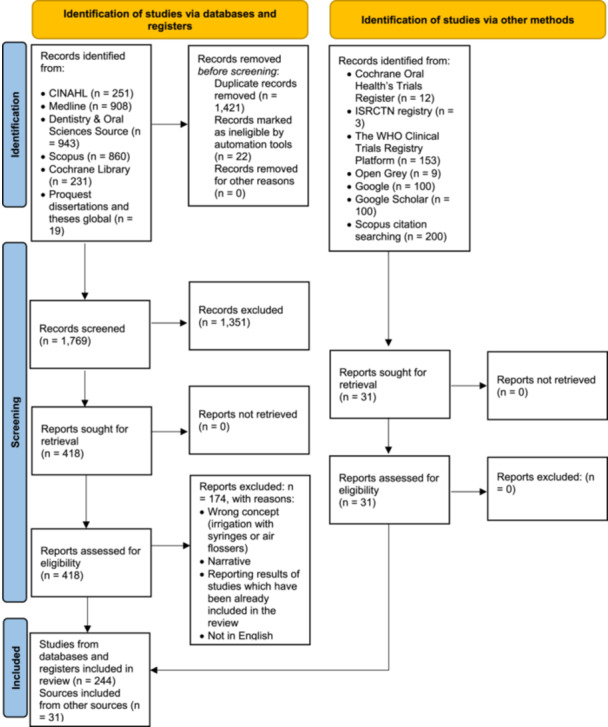
PRISMA flow diagram. Search results and source selection.

Sources identified through databases were uploaded into EndNote VX9 to remove duplicates before being transferred to RAYYAN (Ouzzani et al. 2016; The EndNote Team 2013); 1769 records were kept to screen titles and abstracts. A pilot test involving independent screening of titles and abstracts by two reviewers was conducted to ensure a 75% agreement rate before proceeding with full‐scale screening. Discrepancies were discussed and resolved through discussion. After the titles and abstracts screening, 418 sources were retrieved for the full‐text evaluation. Of those, 174 items did not meet the inclusion criteria, with 244 items included in the review from databases and an additional 31 sources included after the gray literature and Scopus citation searching (Figure 1).

Although we identified several studies referring to air flossers as dental water jets, water flossers, or OIs (Bertl et al. 2022; Kim, Lee, and Jwa 2016; Mazzoleni et al. 2019; Sirinirund et al. 2023; Wiesmüller et al. 2023), these trials were excluded from this scoping review. Unlike conventional OIs, which produce a pressurized water stream, air flossers use a pressured air stream with water droplets (Wiesmüller et al. 2023). Such a significant technological difference calls for differentiation between research on conventional oral irrigation devices and air flossers.

Letters were excluded from the review. However, one letter was selected for inclusion because it presented a case report (Kaplan and Anderson 1977). Furthermore, while narrative reviews were not included in this scoping review, the review by Liang et al. (2020) was selected for inclusion, as the literature search for this evidence‐based practical decision tree was performed systematically.

Data extraction was performed using a refined version of the publicly available data extraction protocol (Sarkisova and Morse 2022). The changes to the data extraction table were based on initial findings to capture all the information necessary to answer the review questions. The aim was to provide a comprehensive overview of the literature, excluding bias risk assessment due to the broad nature of the review. Data were extracted by one reviewer and verified by another, with any disagreements resolved through discussion. We grouped the studies by methodology and further subgrouped them by broad themes, irrigating solutions, and populations.

## 3 Results

### 3.1 Characteristics of the Included Studies

A total of 275 sources were included in the review, predominantly from scientific journals (259) and conducted in academic settings (227), detailed in Supporting Information S1: Appendices 1 and 2.

**Figure 2:**
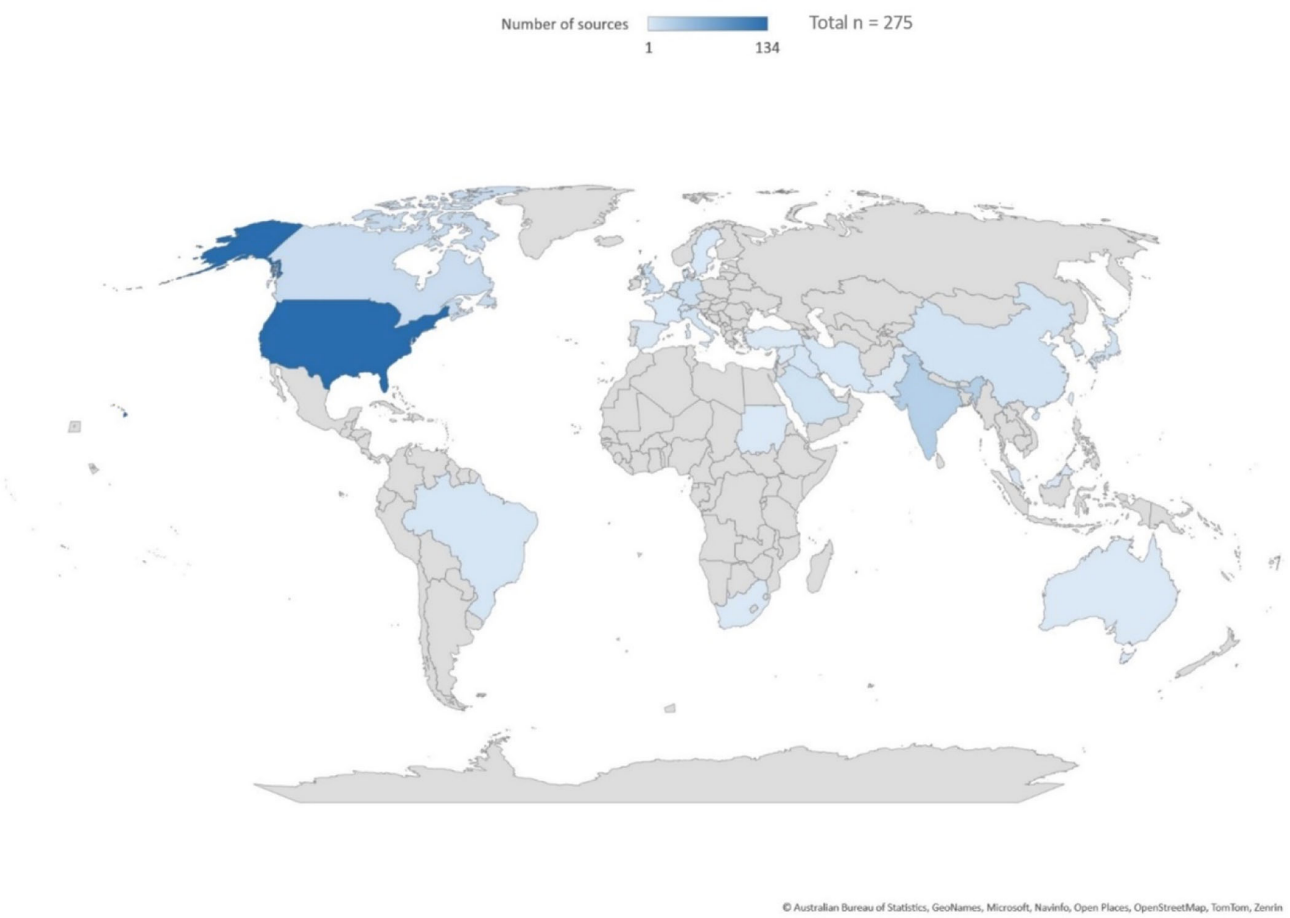
Number of included sources by country (*n* = 275; two sources did not report the location).

**Figure 3:**
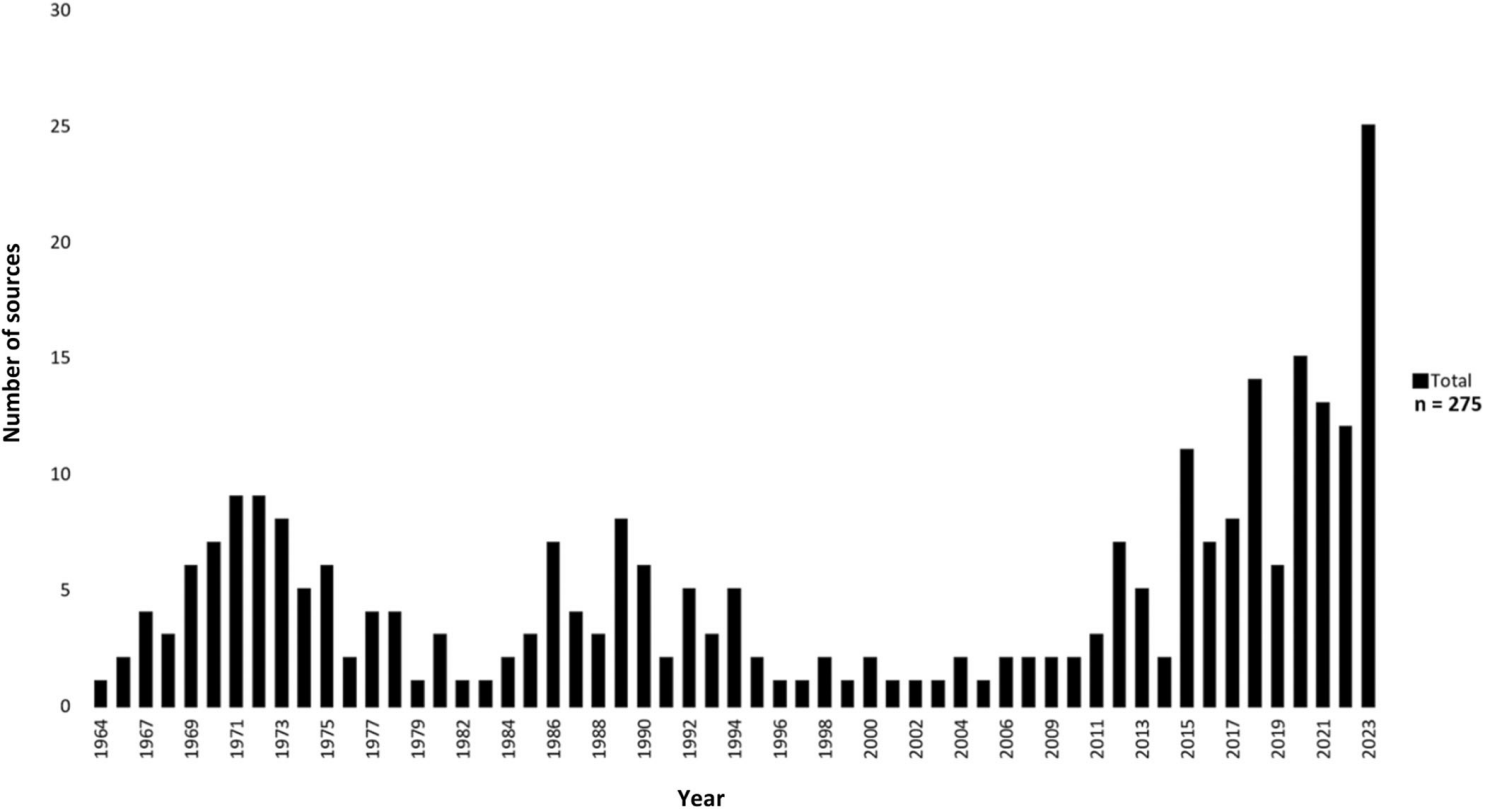
Number of included sources by year (all study types, *n* = 275).

The majority of studies originated from North America (148), followed by Western Europe (49) and Asia (47). Smaller numbers originated from the Middle East (22), South America (4), Africa (2), and Oceania (1), while two did not report location (Figure 2). Publication trends over time are shown in Figure 3.

Regarding study design, 185 were clinical trials, 30 were in vitro studies, 15 systematic reviews, 13 were animal models, 12 were observational studies, 9 were descriptive studies, and 11 described nonoral hygiene applications of OIs (Supporting Information S1: Appendix 3).

Research primarily involved adults, with limited studies on children (6) and adolescents (26). The studies with adolescents often focused on those with fixed orthodontic appliances (15 trials). Children performed self‐irrigation under clinical supervision in only one trial (Murthy et al. 2018).

Industry funding or affiliation was declared in 69 studies (Supporting Information S1: Appendix 4).

#### 3.1.1 Clinical Experimental Studies

The majority of clinical experimental studies were conducted in North America (97), Asia (35), Western Europe (33), and the Middle East (15), with two studies not specifying their location. None were from Eastern Europe or Oceania (Supporting Information S1: Appendix 5).

**Figure 4:**
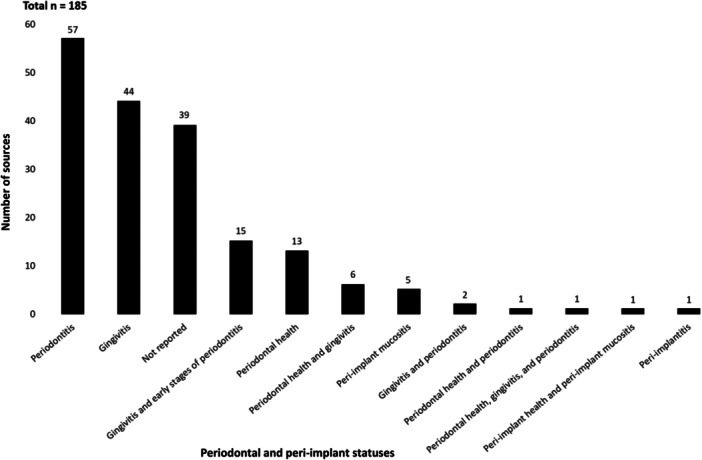
Periodontal and peri‐implant statuses of the populations in the included clinical experimental studies (*n* = 185).

Sample sizes ranged from two to 240 participants, with a mean of 44.1 (SD 36.2). Study durations ranged from a single irrigation episode to 56 weeks. Most trials focused on periodontitis (57), gingivitis (44), or gingivitis and early stages of periodontitis (15). Participants with healthy periodontal status were in 13 studies, a combination of periodontal health and conditions in 8, peri‐implant mucositis in 5, a mix of peri‐implant health and mucositis in 1, and peri‐implantitis in 1; information on periodontal status was not reported in 39 studies (Figure 4).

**Figure 5:**
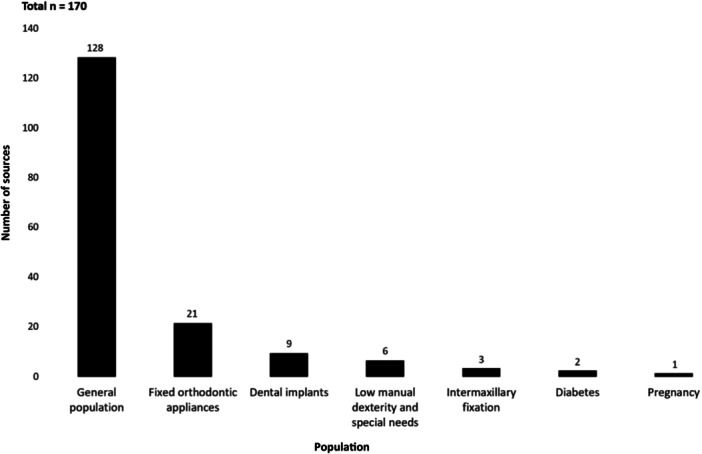
Populations in clinical experimental studies that assessed the effects of oral irrigation on oral hygiene and health (number of studies by population type, *n* = 170).

As shown in Figure 5, most trials (170/185) evaluated oral irrigation effects with various solutions on oral hygiene and oral health in the general population (128), those with fixed orthodontic appliances (21), dental implant recipients (9), special needs groups (6), intermaxillary fixation patients (3), individuals with diabetes (2), and pregnant participants (1 study).

The most common irrigant was water (101). Other solutions tested included antimicrobials other than ozonated water (31), ozonated water (13), herbal extracts (10), and antibiotics compared to antiseptics, water, or saline (9). Specific solutions like acetylsalicylic acid and magnetized water were explored in a few studies (Supporting Information S1: Appendix 6).

Most studies evaluating oral irrigation effectiveness focused only on clinical outcome measures. Indexes used were mainly bleeding, plaque, gingival, periodontal, and calculus. The most commonly used plaque index (37) was the one introduced by Silness and Löe (1964), whereas the index by Löe and Silness (1963) was the most commonly used gingival index (40). Probing depth, clinical attachment loss, and bleeding on probing were also evaluated frequently. Chlorhexidine irrigation staining was assessed in nine trials. In most trials that evaluated the effects of water oral irrigation on clinical outcome measures, OIs were used adjunctively to toothbrushing (69 trials). Only 13 trials had at least one study group that used OIs as a sole oral home care aid. Out of these, irrigation was performed only once in six trials to measure its plaque removal ability.

#### 3.1.2 Systematic Reviews

Fifteen systematic reviews met the inclusion criteria. Of these, only three focused primarily on adjunctive oral irrigation compared to other oral home care aids (Allen 2019; AlMoharib 2023; Husseini, Slot, and Van der Weijden 2008), while the rest reviewed the comparative effectiveness of various oral hygiene aids, including OIs.

Most reviews included studies with healthy adults with gingivitis or periodontitis (Supporting Information S1: Appendix 7). Three assessed home care for dental implants (Bidra et al. 2016; Gennai et al. 2023; Zhao et al. 2022), and two focused on fixed orthodontic appliances (AlMoharib et al. 2023; Pithon et al. 2017).

### 3.2 Descriptive Analysis of the Reported Results

Table 2 summarizes the results of all studies included in the review.

**Table 2:** Summary of the effects of oral irrigation.

Outcome	Summary of the reported results	Number of sources (*n* = 275)^a^
Properties of water sprays	Two zones observed: Impact and flushing zones may aid debris removal	2
Types and brands of devices	Pulsating devices more effective than nonpulsating; faucet‐attached devices could exert higher pressure than self‐contained devices. Waterpik most studied brand (156 sources). Specialized devices for ozonated and magnetized water irrigation	275
Pressure and safety	Excessive pressure risks soft tissue damage and periodontal destruction. Up to 70–90 psi is deemed safe for nonulcerated attached gingiva; for nonkeratinized or ulcerated oral soft tissues, 50–70 psi recommended. Particulate penetration into soft tissue possible	19
Oral irrigation and bacteremia	Bacteremia risk; caution when recommending to individuals at risk of infective endocarditis	10
Effects on dental materials	Generally safe, with some risks to specific composite resins and inlay restorations. Noted increase in surface roughness for microhybrid composites at 100 psi	6
Depth of irrigant penetration	Supragingival tips help reach 44%–71% of pocket depth; subgingival tip potentially reaching the base of pockets	6
Clinical parameters of periodontal and peri‐implant inflammation	Consistent reductions in bleeding, edema, erythema, probing depths, and clinical attachment loss with various irrigants	138
Changes in soft tissue histology and cytology	Reduced inflammation histologically. No adverse implant effects; may enhance osseointegration	12
Dental biofilm	In vitro evidence of biofilm disruption. Clinical and review evidence on plaque reduction mixed. Improved biofilm removal in special needs groups	164
Calculus	Mixed results on calculus prevention	12
Staining	Chlorhexidine increases staining risk. Lower concentrations and subgingival delivery mitigate staining	12
Oral microbiome	Potential shifts away from dysbiosis; further research needed	58
Markers of inflammation	Reductions in select studies; more research warranted	12
GCF volume	Reduction or no significant change	6
Oral irrigators in endodontics	Encouraging in vitro evidence; merits in vivo research	4
Patients' acceptance	Generally high, though studies often lack validated assessment tools	21
Practitioners' and patients' knowledge and attitudes	OIs increasingly recommended by dental practitioners. Low usage rates among the general population	8
Other outcomes	Gingival abrasion: No effect	1
	Halitosis: Mixed results	2
	Capillary wall strength: May increase	1
	Leukocyte chemotactic activity in saliva: May reduce	1
	Calcium and phosphate levels in dental biofilm: No effect	1
	Acidic mouthwash irrigation: Can temporarily reduce oral tissue pH	1
	Alkaline mouthwash irrigation: Can increase pH in the oral cavity	1
	Hydrogen peroxide and sodium hypochlorite irrigation: Reduce drop in plaque pH after a sucrose challenge compared to water irrigation	1
	Water irrigation: No effect on saliva pH	1

Abbreviation: GCF, gingival crevicular fluid.

^a^ Full list of sources is in Supporting Information S1: Appendix 8.

#### 3.2.1 The Qualities of a Water Spray Produced by OIs

Early studies identified two primary zones in the water spray from OIs: A direct impact zone and a flushing zone, highlighting their potential for efficient debris removal (Lugassy and Lautenschlager 1970; Lugassy, Lautenschlager, and Katrana 1971).

#### 3.2.2 Types and Brands of OIs

Waterpik was the most researched brand, used in 139 studies and compared to other brands in 17 studies. Other pulsating and nonpulsating, self‐contained, and faucet‐attached devices were also studied. Faucet‐attached irrigators generated higher forces and were difficult to attach, causing some participants to cease irrigation (Lainson, Bergquist, and Fraleigh 1972; Lugassy, Lautenschlager, and Katrana 1971). All irrigator types could force particulate matter into tissues (O'Leary et al. 1970). Pulsating devices were more effective in reducing inflammation because of the decompression phase (Bhaskar et al. 1971). Faucet‐attached and self‐contained units had similar clinical efficacy (Lainson, Bergquist, and Fraleigh 1970; Oshrain et al. 1987). Specialized devices produced ozonated water (Kent Ozone Aquolab, PURECARE) and magnetized water (Hydro Floss).

#### 3.2.3 Pressure and Safety

Nineteen sources examined irrigation pressure and safety. In vitro, pressures depended on the tip used and could damage tissues (Cutright, Bhaskar, and Larson 1972; Kesavan, Reddy, and Yazdani‐Ardakani 1986; Reddy, Kesavan, and Costarella 1985; Selting and Bhaskar 1973). Excessive pressure caused soft tissue trauma in animals (Bhaskar, Cutright, and Frisch 1969; Cutright et al. 1973). However, 70–90 psi is deemed to be safe for nonulcerated attached gingiva, and 50–70 psi is safe for ulcerated oral soft tissues (Bhaskar et al. 1971). Pressure above 8 g caused pain in inflamed tissue (Lobene 1971). Irrigation has been reported to have caused hemorrhage but no epithelial changes in healthy tissue or in periodontitis (Cobb, Rodgers, and Killoy 1988; Krajewski, Rubach, and Pope 1967). Irrigators had lower pressures than syringes for subgingival use (Kelly et al. 1985). Pressure under 20 psi reduced biofilm (Oshrain et al. 1987), but higher pressures reduced gingival bleeding (Ren et al. 2023).

#### 3.2.4 Oral Irrigation and Bacteremia

Seven studies examined the effects of irrigation on bacteremia. Listerine subgingival irrigation reduced bacteremia after instrumentation in periodontitis (Fine et al. 1996). However, water and chlorhexidine irrigation did not affect bacteremia incidence (Lofthus et al. 1991; Waki et al. 1990). In contrast, irrigation increased bacteremia in those with healthy periodontium (Berger 1974), gingivitis (Romans and App 1971), and periodontitis (Felix, Rosen, and App 1971). Two case reports and one observational study also linked oral irrigation to bacterial endocarditis (Drapkin 1977; Duval et al. 2017; Kaplan and Anderson 1977).

#### 3.2.5 Effects on Dental Materials

Six in vitro studies examined irrigation effects on dental materials over 30 s to 40 years equivalent (Akama et al. 2022; Kotsakis et al. 2021; McDevitt and Eames 1971). Most materials showed no significant surface changes, including composites (Alharbi and Farah 2020), implants (Kotsakis et al. 2021; Matthes et al. 2023), and orthodontic brackets (Akama et al. 2022). However, long‐term irrigation damaged microhybrid composites at 100 psi (Alharbi and Farah 2020), inlay restoration with unacceptable margins (McDevitt and Eames 1971), and bulk fill composites at 63 psi (Naser‐Alavi et al. 2022).

#### 3.2.6 Depth of Subgingival Penetration by Irrigating Solutions

Six studies measured subgingival solution delivery. Irrigant through a supragingival tip reached 44%–71% of pocket depth, with no difference with tip placement at 90° or 45° (Eakle, Ford, and Boyd 1986). Rams and Keyes (1984) first described modifying tips for subgingival use. Subgingival tips delivered to 64%–100% of pocket depth (Braun and Ciancio 1992; Dunkin, Sumner, and Hughes 1989a), and were better than supragingival tips (Larner and Greenstein 1993). An irrigating toothbrush delivered solutions into the pockets more effectively than rinsing (Brackett et al. 2006).

#### 3.2.7 Oral Irrigation and Clinical Parameters of Periodontal and Peri‐Implant Inflammation

A broad range of studies demonstrated oral irrigation's effectiveness in reducing periodontal and peri‐implant inflammation, with significant improvements noted in bleeding, gingival indices, clinical attachment loss, and probing depth. Reduced inflammation was consistently shown in healthy adults, those with diabetes or implants, and children with Papillon–Lefèvre syndrome (3 descriptive and 3 observational studies). Less inflammation occurred with irrigation versus brushing alone or with floss, air flosser, or interdental brushes (119 clinical trials and 13 systematic reviews assessed these outcomes). This was irrespective of the solution, methods, or population. Systematic reviews have reported a reduction in gingival bleeding scores by adjunctive oral water irrigation in patients with gingivitis (Allen 2019; AlMoharib et al. 2023; Edlund et al. 2023; Kotsakis et al. 2018b; Volman, Stellrecht, and Scannapieco 2021); a reduction in gingival index, bleeding scores, and probing depth in periodontitis (Slot, Valkenburg, and Van der Weijden 2020); and a reduction in gingival inflammation in gingivitis and periodontitis (Husseini, Slot, and Van der Weijden 2008; Sälzer et al. 2015; Worthington et al. 2019).

#### 3.2.8 Histological and Cytological Changes

Seven histological and five cytological studies assessed irrigation effects. Irrigation reduced marginal periodontal inflammation but not inflammation in the pocket base (Beget 1967; Cantor and Stahl 1969; Crumley and Sumner 1965; Dunkin 1965; Lainson et al. 1971). It increased keratin layer thickness (Krajewski, Giblink, and Gargiulo 1964), but caused no changes in keratinization patterns (Cantor and Stahl, 1969), epithelial maturation (Covin et al. 1973), or white blood cell counts (Herzog and Hodges 1988). Irrigation enhanced bone formation around dental implants in dogs and preserved implant biocompatibility and osteoblastic growth in vitro (Kotsakis et al. 2021; Matthes et al. 2022; Park et al. 2015).

#### 3.2.9 Oral Irrigation and Biofilm Removal

Numerous studies assessed plaque indices. In vitro studies consistently show efficacy in OIs dislodging biofilm from dental surfaces and restorations, though these outcomes may not replicate clinical experiences. Observational and descriptive research indicates that oral irrigation contributes to lower plaque levels, suggesting a beneficial role in oral hygiene practices (Costa, Costa, and Cota 2020; Hentenaar et al. 2020; Rüdiger, Petersilka, and Flemmig 1999). Clinical trials had conflicting results on natural dentition. Some found no biofilm reduction (23 studies), with a similar number of studies showing improvements over traditional brushing, with or without the addition of floss, air flosser, or interdental brushes (31 studies). Adjunctive irrigation consistently improved biofilm removal in special needs populations (Al‐Mubarak et al. 2002; Deepika et al. 2022; Isshiki 1970). Studies also highlighted effective implant decontamination using oral irrigation with cold atmospheric plasma and Superfloss in vitro and in animal models (Matthes et al. 2023; Park et al. 2015).

Systematic review assessment of the plaque removal ability was not performed because of the high heterogeneity of the included trials (Amarasena, Gnanamanickam, and Miller 2019; Pithon et al. 2017), or found no positive plaque reduction effects (Edlund et al. 2023; Gennai et al. 2023; Husseini, Slot, and Van der Weijden 2008; Kotsakis et al. 2018b; Sälzer et al. 2015; Slot, Valkenburg, and Van der Weijden 2020; Volman, Stellrecht, and Scannapieco 2021; Worthington et al. 2019). Only two reviews showed improvements in plaque scores (Allen 2019; AlMoharib et al. 2023).

#### 3.2.10 Irrigation With Antimicrobial Solutions

Most studies showed significant biofilm reduction with chlorhexidine and ozonated water irrigation. Improvements occurred in individuals with mental disabilities (Tatuskar et al. 2023), pregnant participants (Tecco et al. 2022), orthodontic patients (Dhingra and Vandana 2011; Sandra et al. 2019, 2021), individuals with dental implants (Felo et al. 1997), and intermaxillary fixation patients (Aijima and Yamashita 2023; Soltanianzadeh 2020).

Irrigation with chlorhexidine was more effective in decreasing gingival bleeding in gingivitis and reducing the probing depth and clinical attachment loss in periodontitis compared to rinsing with chlorhexidine mouthwash while also resulting in lower staining scores (Brownstein et al. 1990; Jain et al. 2020). Further, one systematic review reports that irrigation with chlorhexidine is more effective for reducing peri‐implant inflammation in peri‐implant mucositis than rinsing with chlorhexidine mouthwash or applying it as a gel (Zhao et al. 2022). Another potentially clinically relevant solution that effectively reduced biofilm on apatite, metal, and resin material surfaces in vitro is neutral electrolyzed water containing 30 and 100 ppm chlorine (Akama et al. 2022).

#### 3.2.11 Oral Irrigation and Calculus Formation

Twelve studies assessed the effects on calculus. Five found no effect (Gupta, O'Toole, and Hammermeister 1973; Hoover, Robinson, and Billingsley 1968; Lainson, Bergquist, and Fraleigh 1970, 1972; Meklas and Stewart 1972). Three showed decreased calculus with water irrigation (Hoover and Robinson 1971; Kim, Yoo, et al. 2023; Lobene 1969). Benefits occurred with magnetized water (Johnson et al. 1998; Watt, Rosenfelder, and Sutton 1993), and chlorhexidine (Felo et al. 1997). However, one study found increased calculus with chlorhexidine (Flemmig et al. 1990).

#### 3.2.12 Staining

Water and stannous fluoride irrigation did not affect staining (Boyd et al. 1985; Walsh et al. 1989). Chlorhexidine irrigation increased staining in several studies (Agerbaek, Melsen, and Rölla 1975; Flemmig et al. 1990; Lang and Räber 1981; Walsh, Glenwright, and Hull 1992). Watts and Newman (1986) found no staining and lower concentrations of chlorhexidine caused less staining while retaining efficacy (Cumming and Löe 1973; Lang and Ramseier‐Grossmann 1981). Subgingival chlorhexidine irrigation resulted in less staining than rinsing with chlorhexidine mouthwash (Felo et al. 1997; Jain et al. 2020). This may help utilize benefits while mitigating side effects.

#### 3.2.13 Oral Irrigation and Oral Microbiome

Microbiological outcomes were examined in 58 studies using culture, microscopy, polymerase chain reaction, and sequencing. Few assessed periodontitis patients using molecular biology techniques (Deepa et al. 2023; Genovesi et al. 2013; Kshitish and Laxman 2010; Perayil et al. 2016). Overall, oral irrigation reduced pathogens, acidogenic bacteria, anaerobes, spirochetes, and motile rods. Increased periodontal health‐associated species, primary colonizers, and aerobes were reported (Supporting Information S1: Appendix 9).

#### 3.2.14 Oral Irrigation and Markers of Inflammation

Twelve studies on oral irrigation's influence on markers of inflammation showed positive results. Water irrigation did not increase inflammation in vitro (Matthes et al. 2023), or reduce gingival crevicular fluid (GCF) and salivary markers in gingivitis and periodontitis in two trials (Kaur et al. 2023; Ramseier et al. 2021). However, adjunctive water irrigation decreased GCF cytokines versus routine home care (Cutler et al. 2000); adjunctive interdental brushing (Moore et al. 2023); was more effective in participants with peri‐implant mucositis (Tütüncüoğlu et al. 2022); and reduced blood markers in periodontitis and diabetes patients (Al‐Mubarak et al. 2002; Cutler et al. 2000). Ozonated water significantly lowered GCF cytokines (Dhingra and Vandana 2011; Jose et al. 2017; Sandra et al. 2019). Irrigation with water and pomegranate peel extract was comparable in reducing inflammation (Eltay et al. 2021).

#### 3.2.15 GCF Volume

Six studies measured GCF volume changes with irrigation. Most found no effects on GCF flow in gingivitis and periodontitis with various solutions (Ciancio et al. 1989; Ernst et al. 2004; Hugoson 1978; Ravishankar, Venugopal, and Nadkerny 2015). However, pulsated saline irrigation reduced GCF flow more than syringe irrigation in periodontitis (Itic and Serfaty 1992). Ozonated water decreased GCF in orthodontic patients (Dhingra and Vandana 2011).

#### 3.2.16 Oral Irrigation in Special Needs Populations

Oral irrigation showed enhanced biofilm control and reduced gingival inflammation in populations with low manual dexterity and special needs, indicating its utility as an adjunctive or alternative oral hygiene measure in these groups. Notably, modified and cordless irrigators significantly improved oral health outcomes for these populations (Murthy et al. 2018; Yuen 2013; Yuen and Pope 2009). Ozonated water irrigation reduced biofilm, inflammation, and periodontitis‐associated microorganisms in individuals with mental disabilities (Tatuskar et al. 2023).

#### 3.2.17 OIs in Endodontics

The application of OIs for root canal irrigation was studied by two research groups in a total of four in vitro trials (Hemalatha et al. 2022; Shalan and Al‐huwaizi 2017; Shalan, Al‐huwaizi, and Fatalla 2018; Shalan et al. 2018). These studies highlighted OIs' safety and effectiveness in delivering solutions and removing smear layers in root canal systems, suggesting potential applications beyond traditional oral hygiene uses (Hemalatha et al. 2022; Shalan and Al‐huwaizi 2017; Shalan et al. 2018). Although needle choice and placement impacted the apical extrusion of debris and irrigants, overall, the application of OIs resulted in a lower amount of peri‐apically extruded debris and irrigants compared to syringe irrigation and achieved a better smear layer removal efficacy in the apical third of the root canal compared to a sonically driven irrigation system (Shalan and Al‐huwaizi 2017; Shalan, Al‐huwaizi, and Fatalla 2018). The findings from this limited number of in vitro trials call for further in vitro research and clinical validation.

#### 3.2.18 Acceptance, Compliance, and Patient Experiences

There was high satisfaction and compliance with irrigators (Bordabeheres 2012; Flint 2014; Lyle et al. 2020; Peterson and Shiller 1968; Ren et al. 2023; Salles et al. 2021; Sarlati et al. 2016; Sgarbanti et al. 2021). This included populations with fixed orthodontic appliances (Alexander 2006; Schumacher 1978) and tetraplegia (Yuen 2013; Yuen and Pope 2009). However, some patients found irrigation too time‐consuming and messy, and faucet‐attached irrigators were hard to attach (Lainson, Bergquist, and Fraleigh 1972; Peterson and Shiller 1968). Evaluating compliance was difficult if diaries were not returned (Tyler, Kang, and Goh 2023). Clinician‐performed subgingival irrigation (Dunkin, Sumner, and Hughes 1989b), as well as self‐administered supra‐ and subgingival irrigation with various solutions, was well‐tolerated (Flemmig et al. 1990; Walsh, Glenwright, and Hull 1992; Watts and Newman 1986; Wolff et al. 1989).

#### 3.2.19 Practitioners' and Patients' Knowledge and Attitudes

The popularity of oral irrigation devices has been increasing among dental professionals, with recommendations extending to patients with various oral health needs (Hygienetown 2010; Zellmer et al. 2020). In 2020, OIs were the US hygienists' most recommended home care aid for dental implants (Zellmer et al. 2020). Despite this, awareness and usage rates among the general population and nondental practitioners appear low (Stelmakh, Slot, and van der Weijden 2017; Sun et al. 2023; Varela‐Centelles et al. 2020), suggesting an opportunity for broader education and advocacy.

#### 3.2.20 Less Commonly Researched Outcomes

Some studies examined uncommon outcomes. No effect on gingival abrasion was found (Sun et al. 2023). Conflicting results were seen for halitosis improvement (Mishal AlHarbi, Al‐Kadhi, and Al‐Sanea 2020; Xu et al. 2023). Other findings included increased capillary strength (Kozam 1973), reduced salivary leukocyte activity (Wright and Tempel 1974), and variable impacts on oral pH (Aijima and Yamashita 2023; Esposito and Gray 1975; Lobene et al. 1972). No further research followed up on these initial studies.

#### 3.2.21 Applications Other Than Oral Hygiene

In the 1970s, US healthcare providers experimented with pulsating OIs for wound cleaning (Bhaskar et al. 1971; Gross et al. 1971), ophthalmic decontamination (Blumberg 1973), debridement and symptom relief in oral cancer (Bagshaw 1967), kidney stone removal and fecal impaction resolution (Gibbons et al. 1974; Korn 1972), and colonoscopy preparation (Katz et al. 1977). Devices were modified with special attachments in most cases. OIs may help relieve xerostomia symptoms in head and neck cancer patients. This innovative use of OIs was most prominent when the technology first entered the commercial market, with a notable instance of continued interest in the early 2000s (Loud 2001).

#### 3.2.22 Novel and Experimental Oral Irrigators

Some studies focused on experimental devices. Multichannel irrigators with aspiration can be used in bedridden patients and individuals with disabilities, reducing plaque and inflammation (Kim, Yoo, et al. 2023; Kim, Bae et al. 2023). An irrigating toothbrush, which can be used in special needs patients, improved cleaning (Brackett et al. 2006). Experimental oral hygiene need stations were designed to help young children with closed dentition perform interproximal hygiene (Murthy et al. 2018), and improve compliance with interproximal care in people who struggle with interdental cleaning (Fan et al. 2018; Nie 2017).

Other devices focused on subgingival debridement. One creates negative pressure to access deep pockets (Hentenaar et al. 2020; Van Dijk et al. 2018), while others decontaminate implants (Matthes et al. 2022, 2023; Yamada et al. 2017, 2018). An irrigation device delivering antimicrobial gels into periodontal pockets reduced probing depth and bleeding in peri‐implantitis more than regular oral hygiene alone (Levin et al. 2015). While most of these devices are intended for professional in‐office applications, modifying them for at‐home use might be possible.

#### 3.2.23 Research Limitations and Future Directions

In vitro studies were limited by the inability to replicate intraoral conditions, necessitating clinical trials (Abdul Aziz, Afifah, and Syahmi 2018; Ioannidis et al. 2015; Lin, Chuang, and Chang 2018; Matthes et al. 2022, 2023; Tawakoli et al. 2015). Commonly cited clinical limitations included small samples, short durations, lack of blinding, and variability in methods and compliance (Gennai et al. 2023; Volman, Stellrecht, and Scannapieco 2021; Zhao et al. 2022). Future research could include larger trials, longer follow‐ups, comparison of irrigators and air flossers, standardized protocols, clear diagnoses, cost analyses, and patient‐reported outcomes (Amarasena, Gnanamanickam, and Miller 2019; Kotsakis et al. 2018b; Sharma et al. 2012; Worthington et al. 2019).

## 4 Discussion

This scoping review provides a broad, systematic overview of the evidence on oral irrigation devices and identifies research gaps to help direct future research, development, and evidence‐based clinical practice. Oral irrigation was reported as generally safe and well‐accepted when used appropriately as per the manufacturer's guidelines. However, high pressures above 90 psi may cause soft tissue damage (Bhaskar et al. 1971; Lobene 1971; Winter 1982), and the potential for irrigation to cause transient bacteremia indicates caution for individuals at risk of infective endocarditis (Drapkin 1977; Kaplan and Anderson 1977). Overall, evidence indicates that oral irrigation effectively reduces periodontal inflammation, but there are varying results regarding plaque reduction that aligns with systematic reviews (Edlund et al. 2023; Gennai et al. 2023; Husseini, Slot, and Van der Weijden 2008; Kotsakis et al. 2018a; Sälzer et al. 2015; Slot, Valkenburg, and Van der Weijden 2020; Volman, Stellrecht, and Scannapieco 2021; Worthington et al. 2019).

The mechanism underlying anti‐inflammatory effects likely involves shifting oral microbiota away from dysbiosis toward those more consistent with periodontal and dental health rather than completely removing biofilms. Some in vitro evidence showed that oral irrigation disrupted biofilms without fully eliminating them (Brady, Gray, and Bhaskar 1973; Kato, Tamura, and Nakagaki 2012). Furthermore, regular oral irrigation has been reported to result in changes in dental biofilm composition in clinical trials. Ge et al. (2023) observed a reduction in the relative abundance of late colonizers and anaerobic periodontal pathogens, with an increase in early colonizers in the subgingival biofilm of individuals with gingivitis using OIs. A more aerobic phenotype was also observed in supragingival plaque in gingivitis when OIs were used in addition to toothbrushing compared to toothbrushing alone (Xu et al. 2023). Similarly, using multichannel OIs prevented an increase in *Porphyromonas* species in the saliva of individuals with healthy periodontium who discontinued other forms of oral hygiene (Kim, Yoo, et al. 2023). These findings indicate a potential microbiome‐modulating, host‐response–altering mechanism of oral irrigation. Further research using modern molecular biology techniques is needed to clarify the effects on oral microbiota, especially in periodontitis patients where few such studies exist (Deepa et al. 2023; Genovesi et al. 2013; Kshitish and Laxman 2010; Perayil et al. 2016).

Patient acceptance and satisfaction with oral irrigation appear high based on reported experiences (Bordabeheres 2012; Flint 2014; Lyle et al. 2020; Peterson and Shiller 1968; Ren et al. 2023; Salles et al. 2021; Sarlati et al. 2016; Sgarbanti et al. 2021). However, standardized, validated patient‐reported outcome measures were seldom utilized. Incorporating tools quantifying satisfaction, quality‐of‐life impacts, ease of use, and other patient perspectives would strengthen future research (Stelmakh, Slot, and van der Weijden 2017; Sun et al. 2023). Qualitative methods may also help capture patient experiences more comprehensively.

The observed anti‐inflammatory benefits occurred consistently across various populations and irrigant solutions. However, plaque reduction findings were mixed, potentially reflecting differences in study designs, cohorts, devices, follow‐up times, and plaque indices. Standardizing these factors in future trials may improve consistency (Worthington et al. 2019). Controlling for confounders like baseline oral hygiene, pretrial professional plaque removal, specific oral irrigator models, and appropriate statistical analyses would also strengthen reliability.

Evidence in pediatric and special needs populations is sparse but warrants investigation, given oral irrigation's potential in those unable to maintain adequate oral hygiene. Promising results were found in children, physically disabled groups (Isshiki 1970; Yuen 2013), and individuals with mental disabilities (Tatuskar et al. 2023), indicating that feasibility assessments in these cohorts would be justified and clinically valuable if positive. A dearth of research on caries prevention potential needs investigating, as has been previously recommended (Amarasena, Gnanamanickam, and Miller 2019; Worthington et al. 2019).

Sufficiently powered trials with adequate follow‐up periods are required to determine the long‐term effects on periodontal, peri‐implant, and caries outcome measures (Worthington et al. 2019). Achieving sufficient power and follow‐up is particularly challenging in research evaluating caries incidence. Indeed, studies on caries prevention commonly enroll hundreds of participants and require years of follow‐up (Marinho et al. 2013), which may be challenging to achieve with an intervention requiring participant compliance, such as using a home care aid. Furthermore, the results of our review suggest that oral irrigation with water may not be able to remove biofilm completely and is unlikely to affect the pH of dental plaque. Therefore, the successful application of oral irrigation for caries prevention may involve the introduction of irrigation solutions that can significantly reduce biofilm formation or increase biofilm pH without considerable side effects. One possible candidate is electrolyzed water. It is an environmentally friendly and nontoxic solution with potential antiplaque, pH‐neutralizing, and anticariogenic effects (Krishnan et al. 2023). Our review identified one in vitro trial on irrigation with electrolyzed water, and it reported significant antimicrobial and antiplaque results (Akama et al. 2022). Further in vitro and in vivo studies on the application of electrolyzed water as an irrigant for preventing dental caries, periodontal, and peri‐implant diseases may be warranted. Comparing the environmental impacts and costs of using OIs with various irrigants to existing oral hygiene aids would also benefit clinical guidelines and recommendations (Herrera et al. 2023).

Oral irrigation has the potential to be incorporated into oral home care for the secondary prevention of peri‐implantitis, that is, the prevention of disease recurrence in individuals successfully treated for peri‐implantitis. Currently, the EFP clinical practice guideline for the prevention and treatment of peri‐implant diseases suggests that water oral irrigation may be considered an adjunct to regular oral hygiene methods in individuals with peri‐implant mucositis but calls for more research (Herrera et al. 2023). It is also unknown which oral home care method is most effective for individuals treated for peri‐implantitis because of the absence of trials comparing different oral hygiene methods in this population. The results of the present scoping review are consistent with these findings. We identified nine clinical trials that included populations with dental implants, but only one was conducted with a peri‐implantitis population (Levin et al. 2015). Future clinical trials need to assess the effects of oral irrigation on peri‐implantitis and peri‐implant mucositis.

The EFP clinical practice guideline for the treatment of Stage I–III periodontitis suggests that the use of adjunctive chemical agents, particularly chlorhexidine, may be considered in the second (cause‐related) step of periodontal therapy and periodontal maintenance (Sanz et al. 2020). The EFP clinical practice guideline for the prevention and treatment of peri‐implant diseases also supports the time‐limited use of chlorhexidine in individuals with peri‐implant mucositis (Herrera et al. 2023). One important advantage of OIs is their ability to deliver solutions subgingivally (Dunkin, Sumner, and Hughes 1989a; Eakle, Ford, and Boyd 1986). Indeed, OIs are the only oral home care tools that can deliver solutions to the base of deep periodontal pockets (Dunkin, Sumner, and Hughes 1989a). This property of OIs may make them the perfect vehicle for delivering chemical agents in periodontal pockets and peri‐implant sulci. The place of irrigation with antimicrobial solutions in managing periodontitis and peri‐implant mucositis and the possible side effects of such interventions, such as antibiotic crossresistance (Laumen et al. 2021), need to be explored further. In addition, the environmental impact of irrigation with antimicrobials, such as its effects on the water ecosystem and plastic pollution, needs to be studied.

This review has several limitations to consider. Only English‐language sources were included, potentially overlooking research in other languages. No analysis was performed on differences between brands and models. However, as variations in device properties can impact effectiveness, the findings may not be generalizable across all irrigator models. Scoping reviews aim to map the breadth of literature by design, irrespective of the risk of bias or the quality of studies (Tricco et al. 2018). Therefore, the findings must be interpreted cautiously, as no quality assessment was conducted. Further, it is difficult to isolate the effect of oral irrigation because, in the majority of clinical trials, it was used adjunctively to toothbrushing and was compared to an adjunctive use of other interdental cleaning aids, with only a few studies using oral irrigators as the sole oral home care aid. The major strength of this review is that it provides a comprehensive overview of both primary and secondary studies across various regions and settings, providing extensive coverage of the literature over decades.

## 5 Conclusions

Oral irrigation effectively reduces periodontal inflammation, potentially by modulating the oral microbiota. However, knowledge gaps exist regarding its mechanism, effectiveness in plaque removal, impacts on dental caries, patient‐reported outcomes, applicability in pediatric and special needs populations, and environmental and economic considerations. Future trials could address these areas to clarify oral irrigation's role in promoting oral health across diverse populations and conditions.

## Author Contributions

Farzana Sarkisova and Zac Morse were involved in conceptualizing the study; sources identification and screening; data analysis and synthesis; and drafting, revision, and the final approval of the manuscript. Kevin Lee and Nagihan Bostanci contributed to the interpretation of data; revision; and the final approval of the manuscript.

## Ethics Statement

The authors have nothing to report.

## Conflicts of Interest

The authors declare no conflicts of interest.

## Supporting information

Supporting information.

## Data Availability

The data that support the findings of this study are available from the corresponding author upon reasonable request.
