# Supplementary material for: Oral Irrigation Devices: A Scoping Review
Source: Clin Exp Dent Res. 2024 Jun 16;10(3):e912. doi: 10.1002/cre2.912 (PMC11180943; doi:10.1002/cre2.912)
Supplement: Supplementary file 1 — Supporting information. [file CRE2-10-e912-s001.docx]

**Appendix 1.** Types of evidence sources (all sources)

| **Type of source of evidence** | **Number of sources** |
| --- | --- |
| Journal | 259 |
| Repository | 5 |
| Registry | 4 |
| Magazine | 3 |
| Symposium/conference | 2 |
| Database | 1 |
| Manufacturer’s website | 1 |
| Total | 275 |

**Appendix 2**. Research settings of the included sources (all sources)

| **Setting** | **Number of sources** |
| --- | --- |
| Academic | 227 |
| Private research organisation | 19 |
| Military clinic or research organisation | 14 |
| Private clinic | 7 |
| Public clinic | 3 |
| Not reported | 3 |
| Government research organisation | 2 |
| Total | 275 |

**Appendix 3.** Types of the included studies (all sources)

| **Study type** | **Number of sources** |
| --- | --- |
| Clinical experimental studies | 185 |
| In vitro | 30 |
| Reviews | 15 |
| Animal models | 13 |
| Observational | 12 |
| Descriptive – application other than for oral hygiene | 11 |
| Descriptive | 9 |
| Total | 275 |

**Appendix 4.** Sources of funding (all sources)

| **Source of funding** | **Number of sources** |
| --- | --- |
| Not reported | 112 |
| Full or partial industry funding (excluding supply of study materials) or affiliation of one or more researchers with the industry | 69 |
| No specific funding | 32 |
| External, not industry | 25 |
| Internal (institutional) | 14 |
| Source of funding N/R, but the authors declared no conflicts of interest | 12 |
| Unclear (Information on the funding organisation is not publicaly available) | 11 |
| Total | 275 |

**Appendix 5.** Origin of the included clinical experimental studies (number of sources by country)

**
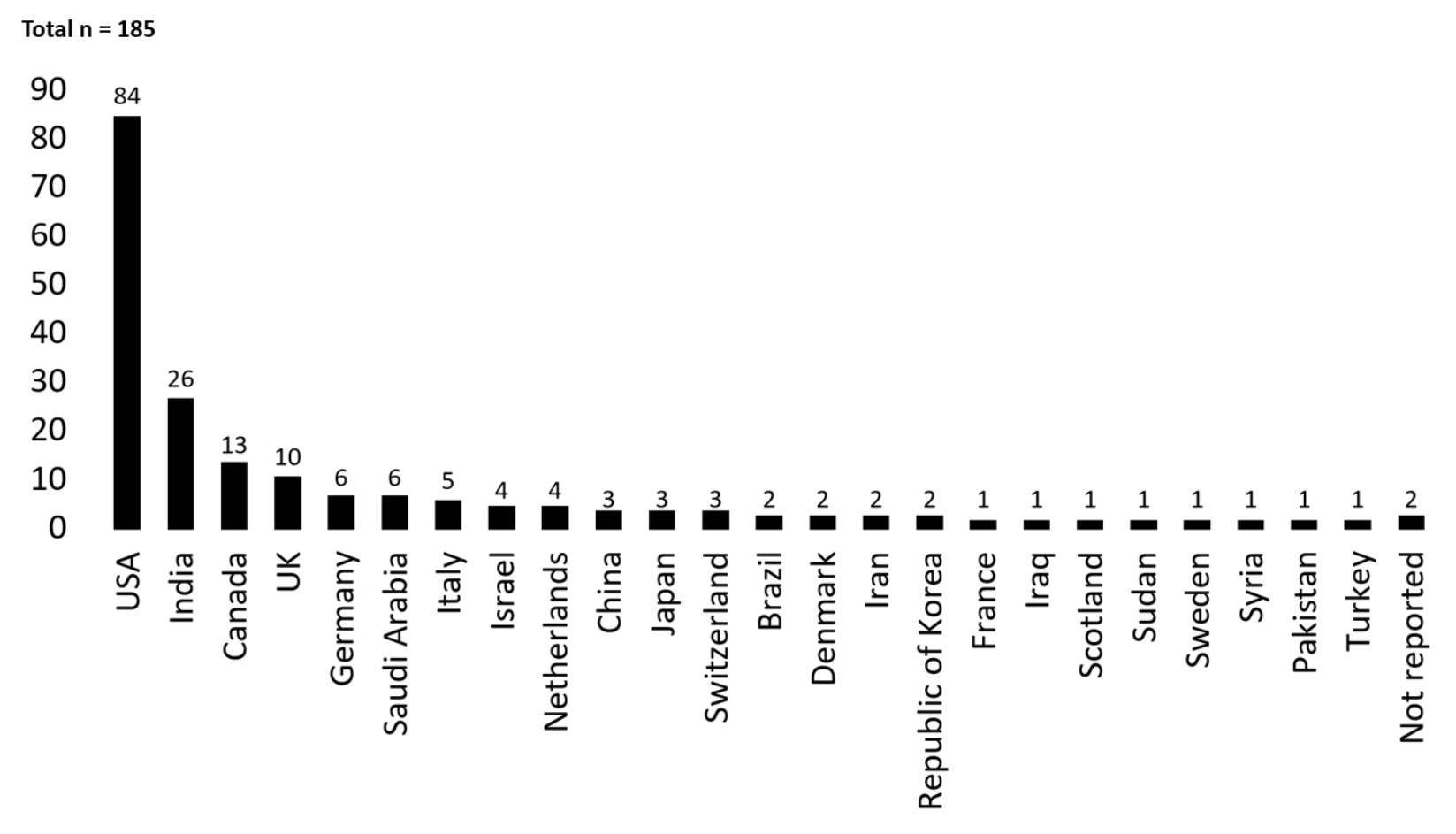
**

**Country**

**Number of sources**

**Appendix 6.** Solutions used for irrigation in clinical trials (total trials n = 185, total comparisons n = 190)

| **Type of irrigant researched in clinical experimental studies** | **Number of sources** |
| --- | --- |
| Water | 101 |
| Antimicrobial solutions (commercially available, non-herbal based, other than ozonated water) vs water, saline, or no irrigation | 31 |
| Saline vs antimicrobial solutions, antibiotic, or no irrigation | 14 |
| Ozonated water | 13 |
| Antibiotics vs antimicrobials, water, or saline | 9 |
| Solutions with dyes and carbon markers | 8 |
| Herbal extracts | 5 |
| Commercially available herbal-based antimicrobial solution | 5 |
| Magnetised water | 2 |
| Acetylsalicylic acid | 1 |

**Appendix 7.** Populations included in the systematic reviews

| **Populations in the review papers** | **Number of sources** |
| --- | --- |
| General population | 10 |
| Dental implants | 3 |
| Fixed orthodontic appliances | 2 |
| Total | 15 |

**Appendix 8.** Full list of studies by the assessed outcomes

| **Outcome** | **Author and year** |
| --- | --- |
| Properties of water sprays (n = 2) | Lugassy and Lautenschlager (1970); Lugassy et al. (1971) |
| Types and brands of devices - Waterpik® (n = 156) | Lugassy and Lautenschlager (1970); Lugassy et al. (1971); McDevitt and Eames (1971); Selting et al. (1972); Selting and Bhaskar (1973); Gorur et al. (2009); Tawakoli et al. (2015); Ioannidis et al. (2015); Ahmed (2019); Alharbi and Farah (2020); Kotsakis et al. (2021); Tardibuono (2021); Naser-Alavi et al. (2022); Hemalatha et al. (2022); Beget (1967); Kancir and Krajewski (1972); Esposito and Gray (1975); Logan (1994); Drapkin (1977); Rams and Keyes (1984); Bordabeheres (2012); Terry et al. (2014); Rams and Slots (2023); Lainson et al. (1972); Tamimi et al. (1969); Felix et al. (1971); Romans and App (1971); Berger et al. (1974); Waki et al. (1990); Lofthus et al. (1991); Kozam (1973); O'Leary et al. (1970); Manhold et al. (1978); Eakle et al. (1986); Dunkin et al. (1989a); Braun and Ciancio (1992); Boyd et al. (1992); Larner and Greenstein (1993); Krajewski et al. (1964); Crumley and Sumner (1965); Krajewski et al. (1967); Tanaka et al. (1968); Peterson and Shiller (1968); Lobene (1969); Cantor and Stahl (1969); Clynes and Wilderman (1970); Fine and Baumhammers (1970); Lainson et al. (1970); Hoover and Robinson (1971); Elliott et al. (1972); AlMoharib et al. (2023); Wright and Tempel (1974); Carter et al. (1975); Tempel et al. (1975); Hugoson (1978); Kelly et al. (1985); Drisko et al. (1987); Oshrain et al. (1987); Cobb et al. (1988); Dunkin et al. (1989b); Itic and Serfaty (1992); Cutler et al. (2000); Barnes et al. (2005); Rosema et al. (2011); Sharma et al. (2012a); Sharma et al. (2012b); Goyal et al. (2012); Genovesi et al. (2013); Goyal et al. (2013); Akram (2015); Goyal et al. (2015); Sasikumar et al. (2016); Lyle et al. (2016); Goyal et al. (2016); Goyal et al. (2018b); Goyal et al. (2018d); Goyal et al. (2018a); Goyal et al. (2018c); Slot et al. (2018); Van Dijk et al. (2018); Lyle et al. (2020); Abdellatif et al. (2021); Batool et al. (2021); Ren et al. (2023); Moore et al. (2023); Agrawal et al. (2023); Behera et al. (2023); Mancinelli-Lyle et al. (2023a); Mancinelli-Lyle et al. (2023b); Ge et al. (2023); Agerbaek et al. (1975); Lang and Räber (1981); Boyd et al. (1985); Watts and Newman (1986); Wolff et al. (1989); Vignarajah et al. (1989); Flemmig et al. (1990); Newman et al. (1990); Brownstein et al. (1990); Jolkovsky et al. (1990); Walsh et al. (1992); Newman et al. (1994); Chaves et al. (1994); Ravindrareddy et al. (2012); Khatri et al. (2017); Jain et al. (2020); Deepa et al. (2023); Aziz-Gandour and Newman (1986); Sanders et al. (1986); Macaulay and Newman (1986); Stabholz et al. (1993); Stabholz et al. (1998); Ravishankar et al. (2015); Perayil et al. (2016); Jalaluddin et al. (2020); Pandya et al. (2016); Kaur et al. (2019); Ciancio et al. (1989); Fine et al. (1994); Fine et al. (1996); Pistorius et al. (2003); Ernst et al. (2004); Abullais et al. (2015); Eltay et al. (2021); Flemmig et al. (1995); Isshiki (1970); Yuen (2013); Deepika et al. (2022); Al-Mubarak et al. (2002); Kaur et al. (2023); Hurst and Madonia (1970); Schumacher (1978); Burch et al. (1994); Alexander (2006); Sharma et al. (2008); Patel et al. (2015); Sandra et al. (2019); Rose et al. (2020); Yeo et al. (2021); Sawan et al. (2022); Tyler et al. (2023); Felo et al. (1997); Magnuson et al. (2013); Bunk et al. (2020); Sgarbanti et al. (2021); Salles, de Cássia Oliveira, Macedo, Silva-Lovato and de Freitas de Oliveira Paranhos (2021); Salles, Oliveira, et al. (2021); Phelps-Sandall and Oxford (1983); Aijima and Yamashita (2023); Bagshaw (1967); Korn (1972); Blumberg (1973); Grossan (1974); Gibbons et al. (1974); Krugman and Beumer (1975); Hoexter (1976); Loud (2001) |
| Types and brands of devices - modified Waterpik® (n = 3) | Bhaskar et al. (1971); Gross et al. (1971); Grower and Bhaskar (1972); |
| Types and brands of devices - Waterpik® used with an ozone generating device (n = 1) | Katti and Chava (2013) |
| Types and brands of devices – a prototype/experimental device (n = 13) | Cutright et al. (1972); Yamada et al. (2017); Yamada et al. (2018); Fan et al. (2018); Abdul Aziz et al. (2018); Bhaskar et al. (1969); Bhaskar et al. (1971); Nie (2017); Lobene (1971); Toto et al. (1969); Listgarten et al. (1989); Jones et al. (1990); Murthy et al. (2018) |
| Types and brands of devices – Kent® ozone (n = 8) | Kshitish and Laxman (2010); Issac et al. (2015); Pandya et al. (2016); Kaur et al. (2019); Tatuskar et al. (2023); Dhingra and Vandana (2011); Jose et al. (2017); Sandra et al. (2021) |
| Types and brands of devices - Aquapick® (n = 6) | Shalan and Al-huwaizi (2017); Shalan, Al-huwaizi, Fatalla, et al. (2018); Shalan, Al-huwaizi and Fatalla (2018); Park et al. (2015); Brook et al. (1977); Soltanianzadeh (2020) |
| Types and brands of devices - Broxojet® (n = 6) | Hock and Tinanoff (1979); Manhold et al. (1978); Walsh et al. (1989); Agerbaek et al. (1975); Lang and Räber (1981); Lang and Ramseier-Grossmann (1981) |
| Types and brands of devices - Dento-Spray® (n = 6) | Lugassy et al. (1971); Beget (1967); O'Leary et al. (1970); Dunkin (1965); Wheatcroft and Sciantarelli (1974); York and Dunkin (1967) |
| Types and brands of devices - Pulsar® (n = 6) | Lugassy et al. (1971); Lainson et al. (1972); Lainson et al. (1970); Lainson et al. (1971); Covin et al. (1973); Gupta et al. (1973) |
| Types and brands of devices – Pro-pulse Viadent® (n = 3) | Herzog and Hodges (1988); Wikesjö et al. (1989); Parsons et al. (1986) |
| Types and brands of devices - Viadent® (n = 3) | Larner and Greenstein (1993); Southard et al. (1987); Parsons et al. (1987) |
| Types and brands of devices – Aquolab® ozone (n = 2) | Tecco et al. (2022); Butera et al. (2021) |
| Types and brands of devices - Aqua Pulse® (n = 2) | Meklas and Stewart (1972); Lobene et al. (1972) |
| Types and brands of devices - Braun® (n = 2) | Frascella et al. (2000); Eberhard et al. (2004) |
| Types and brands of devices - Broxo OraJets® (n = 2) | Caccianiga, Bader, et al. (2022); Caccianiga, Nota, et al. (2022) |
| Types and brands of devices – multichannel COMORAL® (n = 2) | J. Y. Kim et al. (2023); J. M. Kim et al. (2023) |
| Types and brands of devices – an experimental device based on debritom^+^ (n = 2) | Matthes et al. (2022); Matthes et al. (2023) |
| Types and brands of devices - Dentrex® (n = 2) | Kato et al. (2012); Akama et al. (2022) |
| Types and brands of devices - Mouthwasher® (n = 2) | Kancir and Krajewski (1972); O'Leary et al. (1970) |
| Types and brands of devices – Oral B® (n = 2) | Ramseier et al. (2021); Tütüncüoğlu et al. (2022) |
| Types and brands of devices – Woog® (n = 2) | Manhold and Knutsen (1992); Jackson (1991) |
| Types and brands of devices - Aquaflosser® (n = 1) | Ahmed (2019) |
| Types and brands of devices - Aquajet® (n = 1) | Sarlati et al. (2016) |
| Types and brands of devices - Aqua Tec® (n = 1) | Derdivanis et al. (1978) |
| Types and brands of devices - microbubble Braun® with a multichannel tray (n = 1) | Lin et al. (2018) |
| Types and brands of devices - Beizhi® (n = 1) | Wang et al. (2023) |
| Types and brands of devices - Bixdo® (n = 1) | Xu et al. (2023) |
| Types and brands of devices - Fluxion® (n = 1) | Hentenaar et al. (2020) |
| Types and brands of devices – Gum Machine® (n = 1) | Oshrain et al. (1987) |
| Types and brands of devices - Hydrabrush® (n = 1) | Brackett et al. (2006) |
| Types and brands of devices - Hydro-Brush® (n = 1) | Lugassy et al. (1971) |
| Types and brands of devices - Hydro-Dent® (n = 1) | Lugassy et al. (1971) |
| Types and brands of devices - Hydro Floss® (n = 1) | Johnson et al. (1998); Watt et al. (1993) |
| Types and brands of devices – Medical irrigator (n = 1) | Selting and Bhaskar (1973) |
| Types and brands of devices - Olympus Oral Clean® (n = 1) | Lugassy et al. (1971) |
| Types and brands of devices - POH Oral Water Spray® (n = 1) | Lugassy et al. (1971) |
| Types and brands of devices - Pro Jet-stream® (n = 1) | Lugassy et al. (1971) |
| Types and brands of devices - Purecare® (n = 1) | Khaimov and Tobias (2020) |
| Types and brands of devices - Silonite® (n = 1) | Levin et al. (2015) |
| Types and brands of devices - Soshi® (n = 1) | Luo et al. (2023) |
| Types and brands of devices - Water Jet®, JollyDent (n = 1) | Al Hariri et al. (2023) |
| Types and brands of devices - Water Jet®, Northern Electric (n = 1) | Lugassy et al. (1971) |
| Types and brands of devices – Not reported (n = 24) | Cutright et al. (1972); Reddy et al. (1985); Kesavan et al. (1986); Yamada et al. (2017); Yamada et al. (2018); Seliger (1969); Bhaskar et al. (1969); Cutright et al. (1973); Brady et al. (1973); Kaplan and Anderson (1977); Winter (1982); Rüdiger et al. (1999); Lobene (1971); Hoover et al. (1968); Curtis et al. (1988); Flint (2014); Cumming and Löe (1973); Walsh et al. (1995); Krishna et al. (2011); Soni et al. (2012); AlHarbi et al. (2020); AlMoharib (2023); Katz et al. (1977); Diekmann (1984) |
| Pressure and safety (n = 19) | Cutright et al. (1972); Selting and Bhaskar (1973); Reddy et al. (1985); Kesavan et al. (1986); Seliger (1969); Bhaskar et al. (1969); Bhaskar et al. (1971); Kancir and Krajewski (1972); Cutright et al. (1973); Winter (1982); Terry et al. (2014); O'Leary et al. (1970); Manhold et al. (1978); Lobene (1971); Krajewski et al. (1967); Lainson et al. (1971); Kelly et al. (1985); Cobb et al. (1988); Ren et al. (2023) |
| Oral irrigation and bacteraemia (n = 10) | Drapkin (1977); Kaplan and Anderson (1977); Duval et al. (2017); Tamimi et al. (1969); Felix et al. (1971); Romans and App (1971); Berger et al. (1974); Waki et al. (1990); Lofthus et al. (1991); Fine et al. (1996) |
| Effects on dental materials (n = 6) | McDevitt and Eames (1971); Alharbi and Farah (2020); Kotsakis et al. (2021); Akama et al. (2022); Naser-Alavi et al. (2022); Matthes et al. (2023) |
| Depth of irrigant penetration (n = 6) | Rams and Keyes (1984); Eakle et al. (1986); Dunkin et al. (1989a); Braun and Ciancio (1992); Boyd et al. (1992); Larner and Greenstein (1993) |
| Clinical parameters of periodontal inflammation (n = 138) | Rüdiger et al. (1999); Bordabeheres (2012); Rams and Slots (2023); Lainson et al. (1972); Hentenaar et al. (2020); Costa et al. (2020); Crumley and Sumner (1965); Dunkin (1965); Hoover et al. (1968); Lobene (1969); Lainson et al. (1970); Hoover and Robinson (1971); Meklas and Stewart (1972); Covin et al. (1973); Gupta et al. (1973); Wheatcroft and Sciantarelli (1974); Carter et al. (1975); Hugoson (1978); Oshrain et al. (1987); Curtis et al. (1988); Dunkin et al. (1989b); Walsh et al. (1989); Itic and Serfaty (1992); Manhold and Knutsen (1992); Cutler et al. (2000); Frascella et al. (2000); Barnes et al. (2005); Rosema et al. (2011); Sharma et al. (2012b); Goyal et al. (2012); Genovesi et al. (2013); Flint (2014); Akram (2015); Goyal et al. (2015); Sasikumar et al. (2016); Goyal et al. (2016); Goyal et al. (2018b); Goyal et al. (2018d); Goyal et al. (2018a); Goyal et al. (2018c); Slot et al. (2018); Van Dijk et al. (2018); Lyle et al. (2020); Ramseier et al. (2021); Caccianiga, Bader, et al. (2022); Ren et al. (2023); Moore et al. (2023); Xu et al. (2023); Agrawal et al. (2023); Mancinelli-Lyle et al. (2023a); Mancinelli-Lyle et al. (2023b); J. Y. Kim et al. (2023); J. M. Kim et al. (2023); Lobene et al. (1972); Lang and Räber (1981); Lang and Ramseier-Grossmann (1981); Boyd et al. (1985); Watts and Newman (1986); Herzog and Hodges (1988); Listgarten et al. (1989); Wolff et al. (1989); Vignarajah et al. (1989); Jones et al. (1990); Flemmig et al. (1990); Brownstein et al. (1990); Jolkovsky et al. (1990); Walsh et al. (1992); Newman et al. (1994); Chaves et al. (1994); Ravindrareddy et al. (2012); Khatri et al. (2017); Jain et al. (2020); Deepa et al. (2023); Aziz-Gandour and Newman (1986); Stabholz et al. (1998); Krishna et al. (2011); Ravishankar et al. (2015); Perayil et al. (2016); Jalaluddin et al. (2020); Kshitish and Laxman (2010); Katti and Chava (2013); Issac et al. (2015); Pandya et al. (2016); Kaur et al. (2019); Khaimov and Tobias (2020); Southard et al. (1987); Parsons et al. (1986); Parsons et al. (1987); Ciancio et al. (1989); Fine et al. (1994); Pistorius et al. (2003); Ernst et al. (2004); Abullais et al. (2015); Eltay et al. (2021); Flemmig et al. (1995); Johnson et al. (1998); Yuen (2013); Deepika et al. (2022); Tatuskar et al. (2023); Al-Mubarak et al. (2002); Tecco et al. (2022); Kaur et al. (2023); York and Dunkin (1967); Schumacher (1978); Jackson (1991); Burch et al. (1994); Alexander (2006); Sharma et al. (2008); Dhingra and Vandana (2011); Soni et al. (2012); Patel et al. (2015); Jose et al. (2017); Sandra et al. (2019); Sandra et al. (2021); Tyler et al. (2023); AlMoharib (2023); Felo et al. (1997); Magnuson et al. (2013); Levin et al. (2015); Bunk et al. (2020); Sgarbanti et al. (2021); Butera et al. (2021); Salles, de Cássia Oliveira, Macedo, Silva-Lovato and Paranhos (2021); Tütüncüoğlu et al. (2022); Phelps-Sandall and Oxford (1983); Soltanianzadeh (2020); Husseini et al. (2008); Sälzer et al. (2015); Bidra et al. (2016); Kotsakis et al. (2018); Worthington et al. (2019); Amarasena et al. (2019); Allen (2019); Slot et al. (2020); Volman et al. (2021); Zhao et al. (2022); Gennai et al. (2023); Edlund et al. (2023); AlMoharib et al. (2023) |
| Changes in soft tissue histology (n = 7) | Beget (1967); Park et al. (2015); Krajewski et al. (1964); Crumley and Sumner (1965); Dunkin (1965); Cantor and Stahl (1969); Lainson et al. (1971) |
| Changes in soft tissue cytology (n = 5) | Kotsakis et al. (2021); Matthes et al. (2022); O'Leary et al. (1970); Covin et al. (1973); Herzog and Hodges (1988) |
| Dental biofilm (n = 164) | Selting et al. (1972); Gorur et al. (2009); Kato et al. (2012); Tawakoli et al. (2015); Ioannidis et al. (2015); Yamada et al. (2017); Yamada et al. (2018); Abdul Aziz et al. (2018); Ahmed (2019); Kotsakis et al. (2021); Akama et al. (2022); Matthes et al. (2023); Wang et al. (2023); Brady et al. (1973); Hock and Tinanoff (1979); Park et al. (2015); Logan (1994); Rüdiger et al. (1999); Lainson et al. (1972); Silver and Chesko (1975); Hentenaar et al. (2020); Costa et al. (2020); Krajewski et al. (1964); Dunkin (1965); Hoover et al. (1968); Peterson and Shiller (1968); Lobene (1969); Toto et al. (1969); Clynes and Wilderman (1970); Fine and Baumhammers (1970); Lainson et al. (1970); Hoover and Robinson (1971); Elliott et al. (1972); Meklas and Stewart (1972); Covin et al. (1973); Gupta et al. (1973); Wheatcroft and Sciantarelli (1974); Tempel et al. (1975); Hugoson (1978); Oshrain et al. (1987); Walsh et al. (1989); Itic and Serfaty (1992); Manhold and Knutsen (1992); Cutler et al. (2000); Frascella et al. (2000); Eberhard et al. (2004); Barnes et al. (2005); Rosema et al. (2011); Sharma et al. (2012a); Sharma et al. (2012b); Goyal et al. (2012); Genovesi et al. (2013); Goyal et al. (2013); Akram (2015); Goyal et al. (2015); Sasikumar et al. (2016); Sarlati et al. (2016); Lyle et al. (2016); Goyal et al. (2016); Goyal et al. (2018b); Goyal et al. (2018a); Goyal et al. (2018c); Van Dijk et al. (2018); Lyle et al. (2020); Ramseier et al. (2021); Abdellatif et al. (2021); Batool et al. (2021); Caccianiga, Bader, et al. (2022); Ren et al. (2023); Moore et al. (2023); Xu et al. (2023); Luo et al. (2023); Agrawal et al. (2023); Behera et al. (2023); Mancinelli-Lyle et al. (2023a); Mancinelli-Lyle et al. (2023b); J. Y. Kim et al. (2023); J. M. Kim et al. (2023); Cumming and Löe (1973); Agerbaek et al. (1975); Derdivanis et al. (1978); Lang and Räber (1981); Lang and Ramseier-Grossmann (1981); Boyd et al. (1985); Watts and Newman (1986); Listgarten et al. (1989); Wolff et al. (1989); Vignarajah et al. (1989); Jones et al. (1990); Flemmig et al. (1990); Brownstein et al. (1990); Jolkovsky et al. (1990); Walsh et al. (1992); Newman et al. (1994); Chaves et al. (1994); Ravindrareddy et al. (2012); Khatri et al. (2017); Jain et al. (2020); Deepa et al. (2023); Aziz-Gandour and Newman (1986); Stabholz et al. (1998); Krishna et al. (2011); Ravishankar et al. (2015); Perayil et al. (2016); Jalaluddin et al. (2020); Kshitish and Laxman (2010); Katti and Chava (2013); Kaur et al. (2019); Southard et al. (1987); Parsons et al. (1986); Parsons et al. (1987); Ciancio et al. (1989); Fine et al. (1994); Pistorius et al. (2003); Ernst et al. (2004); Abullais et al. (2015); Eltay et al. (2021); Flemmig et al. (1995); Johnson et al. (1998); Isshiki (1970); Murthy et al. (2018); Deepika et al. (2022); Tatuskar et al. (2023); Al-Mubarak et al. (2002); Tecco et al. (2022); Kaur et al. (2023); York and Dunkin (1967); Schumacher (1978); Jackson (1991); Burch et al. (1994); Alexander (2006); Sharma et al. (2008); Dhingra and Vandana (2011); Soni et al. (2012); Patel et al. (2015); Sandra et al. (2019); Rose et al. (2020); Sandra et al. (2021); Sawan et al. (2022); Tyler et al. (2023); Al Hariri et al. (2023); AlMoharib (2023); Felo et al. (1997); Bunk et al. (2020); Sgarbanti et al. (2021); Butera et al. (2021); Salles, de Cássia Oliveira, Macedo, Silva-Lovato and Paranhos (2021); Tütüncüoğlu et al. (2022); Phelps-Sandall and Oxford (1983); Soltanianzadeh (2020); Aijima and Yamashita (2023); Husseini et al. (2008); Sälzer et al. (2015); Pithon et al. (2017); Kotsakis et al. (2018); Worthington et al. (2019); Amarasena et al. (2019); Allen (2019); Slot et al. (2020); Volman et al. (2021); Zhao et al. (2022); Gennai et al. (2023); Edlund et al. (2023); AlMoharib et al. (2023) |
| Calculus (n = 12) | Lainson et al. (1972); Hoover et al. (1968); Lobene (1969); Lainson et al. (1970); Hoover and Robinson (1971); Meklas and Stewart (1972); Gupta et al. (1973); J. M. Kim et al. (2023); Flemmig et al. (1990); Johnson et al. (1998); Felo et al. (1997); Watt et al. (1993) |
| Staining (n = 12) | Walsh et al. (1989); Cumming and Löe (1973); Agerbaek et al. (1975); Lang and Räber (1981); Lang and Ramseier-Grossmann (1981); Boyd et al. (1985); Watts and Newman (1986); Flemmig et al. (1990); Walsh et al. (1992); Jain et al. (2020); Kaur et al. (2023); Felo et al. (1997) |
| Oral microbiome (n = 58) | Refer to Appendix 9 (Effects of oral irrigation on oral microbiome) |
| Markers of inflammation (n = 12) | Matthes et al. (2022); Cutler et al. (2000); Ramseier et al. (2021); Moore et al. (2023); Eltay et al. (2021); Al-Mubarak et al. (2002); Kaur et al. (2023); Dhingra and Vandana (2011); Jose et al. (2017); Sandra et al. (2019); Sandra et al. (2021); Tütüncüoğlu et al. (2022) |
| GCF^†^ volume (n = 6) | Hugoson (1978); Itic and Serfaty (1992); Ravishankar et al. (2015); Ciancio et al. (1989); Ernst et al. (2004); Dhingra and Vandana (2011) |
| Oral irrigators in endodontics (n = 4) | Shalan and Al-huwaizi (2017); Shalan, Al-huwaizi, Fatalla, et al. (2018); Shalan, Al-huwaizi and Fatalla (2018); Hemalatha et al. (2022) |
| Patients’ acceptance (n = 21) | Bordabeheres (2012); Lainson et al. (1972); Peterson and Shiller (1968); Dunkin et al. (1989b); Flint (2014); Sarlati et al. (2016); Lyle et al. (2020);Ren et al. (2023); J. Y. Kim et al. (2023); Boyd et al. (1985); Watts and Newman (1986); Flemmig et al. (1990); Wolff et al. (1989); Walsh et al. (1992); Yuen and Pope (2009); Yuen (2013); Schumacher (1978); Alexander (2006); Tyler et al. (2023); Sgarbanti et al. (2021); Salles, de Cássia Oliveira, Macedo, Silva-Lovato and Paranhos (2021) |
| Practitioners’ and patients’ knowledge and attitudes (n = 8) | Silver and Chesko (1975); Gosnell (1981); Hygienetown (2010, May); Stelmakh et al. (2017); Zellmer et al. (2020); Varela-Centelles et al. (2020); ElShiekh et al. (2022); Sun et al. (2023) |
| Gingival abrasion (n = 1) | Slot et al. (2018) |
| Halitosis (n = 2) | Xu et al. (2023); AlHarbi et al. (2020) |
| Capillary wall strength (n = 1) | Kozam (1973) |
| Leukocyte chemotactic activity in saliva (n = 1) | Wright and Tempel (1974) |
| Calcium and phosphate levels in dental biofilm (n = 1) | Brook et al. (1977); Sun et al. (2023) |
| pH of the oral tissues - Acidic mouthwash irrigation (n = 1) | Esposito and Gray (1975) |
| pH of the oral tissues - Alkaline mouthwash irrigation (n = 1) | Esposito and Gray (1975) |
| Plaque pH - Hydrogen peroxide and sodium hypochlorite irrigation (n = 1) | Lobene et al. (1972) |
| Saliva pH - Water irrigation (n = 1) | Aijima and Yamashita (2023) |

^†^GCF – Gingival crevicular fluid

**Appendix 9.** Effects of oral irrigation on oral microbiome

| **Study type** | **Technique** | **Author and year** | **Periodontal status** | **Reported effects of oral irigation** |
| --- | --- | --- | --- | --- |
| In vitro | Culture | Tawakoli et al. (2015); Ioannidis et al. (2015); Lin et al. (2018); Kotsakis et al. (2021); Tardibuono (2021); Akama et al. (2022); Matthes et al. (2023); Wang et al. (2023); Katti and Chava (2013) | N/A | **Water –** ↓ bacterial count  **Water with air bubbles** **–** ↓ facultative anaerobes  **Water + cold atmospheric pressure plasma –** ↓ bacterial count  **Neutral electrolysed water containing chlorine –** ↓ bacterial count  **Normal saline** – ↓ *Streptococcus mutans*  **Chlorhexidine** **–** ↓ bacterial count  **Ozonated water** – ↓red complex |
| Animal models |  | Bhaskar et al. (1971) | N/A | **Water** – ↓ bacteria in contaminated wounds, addition of antibiotics, particularly a mixture of **streptomycin** and **penicillin**, enhances antibacterial effects of irrigation |
| Descriptive | Culture | Rams and Slots (2023) | Periodontitis | **Sodium hypochlorite** – ↓ periodontal pathogens, ↑ species associated with periodontal health |
|  | qPCR^†^ | Rüdiger et al. (1999) | Periodontitis | **Chlorhexidine** – suppression of *Aggregatibacter actinomycetemcomitans* |
| Observational | qPCR | Hentenaar et al. (2020) | Peri-implantitis | **Water** – No significant effect |
| Clinical experimental | Culture | Tanaka et al. (1968); Toto et al. (1969); Wikesjö et al. (1989); Newman et al. (1990); Brownstein et al. (1990); Jolkovsky et al. (1990); Chaves et al. (1994); Stabholz et al. (1993); Jalaluddin et al. (2020); Kshitish and Laxman (2010); Issac et al. (2015); Pandya et al. (2016); Fine et al. (1994); Tatuskar et al. (2023); Hurst and Madonia (1970) | Gingivitis – 3  Periodontitis – 9  Gingivitis and periodontitis – 1  N/R – 3 | **Water** – ↓ anaerobes, acidogenic bacteria, *Prevotella intermedia,* periodontitis-associated bacteria  **Hydrogen peroxide** – ↓ *A. actinomycetemcomitans*  **Chlorhexidine** – ↓ Gram-negative anaerobic rods, black-pigmented *Bacteroides, Actinomyces, Fusobacteria, Wolinella recta, Prevotella intermedia, Porphyromonas gingivalis*  **Tetracycline** – significant antimicrobial activity, ↓ *P. gingivalis*  **Povidone-iodine** – ↓ *P. gingivalis*  **Ozonated water** – ↓ anaerobes, *P. intermedia*, *P. gingivalis*, and *Fusobacterium nucleatum*  **Listerine®** – ↓ periodontitis-associated bacteria, ↑ coccoid cells |
|  | Microscopy | Dunkin (1965); Fine and Baumhammers (1970); Drisko et al. (1987); Cobb et al. (1988); Itic and Serfaty (1992); Caccianiga, Bader, et al. (2022); Derdivanis et al. (1978); Boyd et al. (1985); Herzog and Hodges (1988); Listgarten et al. (1989); Walsh et al. (1995); Sanders et al. (1986); Macaulay and Newman (1986); Stabholz et al. (1998); Krishna et al. (2011); Ciancio et al. (1989); Fine et al. (1994); Abullais et al. (2015); York and Dunkin (1967); Caccianiga, Nota, et al. (2022) | Health – 1  Health and periodontitis – 1  Gingivitis and periodontitis – 1  Periodontitis – 15  N/R – 2 | **Water** – Restoration of healthy microflora; ↓ bacterial mass, periodontal pathogens, motile bacteria, spirochetes; ↓ spirochetes, fusiform, and branching microorganisms in deep pockets. No effect on Gram-staining properties of biofilm  **Herbal extract and chlorhexidine** - similar spirochete counts  **Listerine®** – ↓ periodontitis-associated bacteria, bacterial cell counts, motile rods, ↑ coccoid cells  **Tetracycline** – ↓ spirochetes and motile rods, ↑ coccoid cells  **Metronidazole** – ↓ motile organisms  **Chlorhexidine** – ↓ motile organisms, vitality of microorganisms  **Saline** (a single irrigation) – no effect  **Tetrapotassium peroxydiphosphate** – ↓ spirochetes and motile rods, ↑ coccoid cells  **Chloramine-T** –↓ spirochetes  **Stannous fluoride** – ↓ spirochetes and motile rods, ↑ coccoid cells  **Cetylpyridinium chloride** – ↓ plaque maturation |
|  | PCR^‡^ techniques | Genovesi et al. (2013); Xu et al. (2023); Luo et al. (2023); Ge et al. (2023); Deepa et al. (2023); Perayil et al. (2016); Kshitish and Laxman (2010); Yeo et al. (2021) | Periodontal health and gingivitis – 1  Gingivitis – 2  Periodontitis – 4  N/R – 1 | **Water irrigation** is effective for preventing re-colonisation by periodontal pathogens; shifts microbiota to less anaerobic phenotype, ↓ late colonisers, genus *Prevotella* and species *P. intermedia*, *S. mutans*  ↑ genus *Corynebacterium,* early colonisers (*Streptococcus, Veillonella, and Fusobacterium*).  **Sodium hypochlorite** – ↓ red complex  **Tetracycline** and **povidone-iodine** – ↓ *P. gingivalis* and *Tannerella forsythia*  **Ozonated** **water** – ↓ *A. actinomycetemcomitans;* an antifungal effect  **Chlorhexidine** – antiviral effect  **Hexamidine** – ↓ red complex |
|  | DNA-DNA hybridisation | Salles, Oliveira, et al. (2021) | N/R, performed on edentulous individuals with mandibular overdentures retained by implants or mini-implants | **Water** *– ↓ Campylobacter rectus, Porphyromonas endodontalis, Veillonella parvula, A. actinomycetemcomitans, Bacteroides fragilis, Enterococcus faecalis, Lactobacillus casei, Staphylococcus pasteuri, Streptococcus constellatus,* and *S. mutans* in the subgingival sulci biofilm |
|  | Sequencing | Xu et al. (2023); Ge et al. (2023); J. M. Kim et al. (2023) | Health – 1  Gingivitis – 2 | **Water** – ↑ *Streptococcus gordonii,* genus *Corynebacterium,* commensals and early colonisers (*Streptococci*, *Veillonellae,* and *Fusobacteria*), a shift towards less anaerobic microflora, *↓* genus *Prevotella* and species *P. intermedia,* late colonisers |
|  | Not reported | J. Y. Kim et al. (2023) | Health and gingivitis | **Water** – No significant effect |

^†^qPCR – quantitative polymerase chain reaction; ^‡^PCR – polymerase chain reaction

References

Abdellatif, H., Alnaeimi, N., Alruwais, H., Aldajan, R., & Hebbal, M. I. (2021). Comparison between water flosser and regular floss in the efficacy of plaque removal in patients after single use. *The Saudi Dental Journal, 33*(5), 256–259. <https://doi.org/10.1016/j.sdentj.2021.03.005>

Abdul Aziz, A. H., Afifah, N. A., & Syahmi, N. M. (2018). New water irrigator for cleaning dental plaque. *International Journal for Innovation Education and Research, 6*(10), 299–305. <https://doi.org/10.31686/ijier.Vol6.Iss10.1190>

Abullais, S. S., Dani, N., Hamiduddin, P. N., Kudyar, N., & Gore, A. (2015). Efficacy of irrigation with different antimicrobial agents on periodontal health in patients treated for chronic periodontitis: A randomized controlled clinical trial. *Ayu, 36*(4), 380–386. <https://doi.org/10.4103/0974-8520.190702>

Agerbaek, N., Melsen, B., & Rölla, G. (1975). Application of chlorhexidine by oral irrigation systems. *Scandinavian Journal of Dental Research, 83*(5), 284–287. <https://doi.org/10.1111/j.1600-0722.1975.tb00439.x>

Agrawal, A., Sawhney, A., Panda, S., Gupta, N., Amol Khale, P., Rathod, V., & Singh Makkad, R. (2023). Comparison of the efficacy of different oral hygiene aids in maintaining periodontal health in patients with gingivitis. *Cureus, 15*(8), Article e44391. <https://doi.org/10.7759/cureus.44391>

Ahmed, O. (2019). *An in-vitro evaluation of the efficacy of oral devices to remove dental biofilm from three prosthodontic materials* [Master's thesis, University of the Western Cape]. The University of the Western Cape Electronic Theses and Dissertations Repository. <https://etd.uwc.ac.za/handle/11394/6635>

Aijima, R., & Yamashita, Y. (2023). Effectiveness of perioperative oral hygiene management using a cetylpyridinium chloride‐, dipotassium glycyrrhizinate, and tranexamic acid‐based mouthwash as an adjunct to mechanical oral hygiene in patients with maxillomandibular fixation: A randomized controlled clinical trial. *Clinical and Experimental Dental Research, 9*(6), 1044–1050. <https://doi.org/10.1002/cre2.814>

Akama, Y., Nagamatsu, Y., Ikeda, H., Nakao-Kuroishi, K., Kometani-Gunjigake, K., Kawamoto, T., & Shimizu, H. (2022). Applicability of neutral electrolyzed water for cleaning contaminated fixed orthodontic appliances. *American Journal of Orthodontics and Dentofacial Orthopedics, 161*(6), 507–523. <https://doi.org/10.1016/j.ajodo.2022.02.007>

Akram, H. M. (2015). Comparison between powerful Waterpik flosser with dental floss as an adjunct to tooth brushing. *Journal of Baghdad College of Dentistry, 27*(3), 89–92.

Al-Mubarak, S., Ciancio, S., Aljada, A., Mohanty, P., Ross, C., & Dandona, P. (2002). Comparative evaluation of adjunctive oral irrigation in diabetics. *Journal of Clinical Periodontology, 29*(4), 295–300. <https://doi.org/10.1034/j.1600-051x.2002.290404.x>.

Al Hariri, M. H., Karkoutly, M., Al Kurdi, S., Alkassar, M., & Bshara, N. (2023). The efficacy of the dental water jet, orthodontic, and conventional toothbrushes in plaque removal around orthodontic braces in adolescents: A randomized controlled trial. *Clinical and Experimental Dental Research, 9*(4), 606–613. <https://doi.org/10.1002/cre2.752>

Alexander, K. M. (2006). *A clinical evaluation of the effects of oral irrigation on the gingival health of adult orthodontic patients* [Master of Science, The State University of New York at Buffalo]. ProQuest Dissertations & Theses Global. <https://www.proquest.com/docview/304940501?pq-origsite=gscholar&fromopenview=true&sourcetype=Dissertations%20&%20Theses>

Alharbi, M., & Farah, R. (2020). Effect of water-jet flossing on surface roughness and color stability of dental resin-based composites. *Journal of Clinical and Experimental Dentistry, 12*(2), e169–e177. <https://doi.org/10.4317/jced.56153>

AlHarbi, M. A., Al-Kadhi, O., & Al-Sanea, J. (2020). The efficacy of different methods of teeth cleaning on the levels of volatile sulfur compounds in patients with orthodontic fixed appliances. *Oral Health Care, 5*, 1–8. <https://doi.org/10.15761/OHC.1000196>

Allen, J. (2019). Efficacy of oral irrigators on gingival health: A review of three papers. *Dental Health, 58*(1), 34–40.

AlMoharib, H. S. (2023). *The effectiveness of water jet flossing and interdental flossing for oral hygiene in orthodontic patients with fixed appliances: A randomised clinical trial*. Thai Clinical Trials Registry. <https://www.thaiclinicaltrials.org/show/TCTR20230926005>

AlMoharib, H. S., AlAskar, M. H., AlShabib, A. N., Almadhoon, H. W., & AlMohareb, T. S. (2023). The effectiveness of dental water jet in reducing dental plaque and gingival bleeding in orthodontic patients: A systematic review and meta-analysis of randomized clinical trials. *International Journal of Dental Hygiene, 22*(1), 56–64. <https://doi.org/10.1111/idh.12741>

Amarasena, N., Gnanamanickam, E. S., & Miller, J. (2019). Effects of interdental cleaning devices in preventing dental caries and periodontal diseases: A scoping review. *Australian Dental Journal, 64*(4), 327–337. <https://doi.org/10.1111/adj.12722>

Aziz-Gandour, I. A., & Newman, H. N. (1986). The effects of a simplified oral hygiene regime plus supragingival irrigation with chlorhexidine or metronidazole on chronic inflammatory periodontal disease. *Journal of Clinical Periodontology, 13*(3), 228–236. <https://doi.org/10.1111/j.1600-051x.1986.tb01465.x>

Bagshaw, M. A. (1967). A water-jet device for supportive care in oral cancer. *The American Journal of Roentgenology, Radium Therapy, and Nuclear Medicine, 99*(4), 842. <https://doi.org/10.2214/ajr.99.4.842>

Barnes, C. M., Russell, C. M., Reinhardt, R. A., Payne, J. B., & Lyle, D. M. (2005). Comparison of irrigation to floss as an adjunct to tooth brushing: Effect on bleeding, gingivitis, and supragingival plaque. *The Journal of Clinical Dentistry, 16*(3), 71–77.

Batool, S. M., Rasheed, M., Syed, K., Farooq, A., Ayub, Z., & Raheem, A. (2021). Plaque removal efficacy of dental floss compare to water jet following single use. *Pakistan Journal of Medical and Health Sciences, 15*(12), 3439–3441. <https://doi.org/10.53350/pjmhs2115123439>

Beget, B. C. (1967). Oral irrigation and inflammation [Abstract]. International Association for Dental Research, Washington, USA.

Behera, R. R., Jalaluddin, M., Kalgeri, S. H., Mailankote, S., Penumatsa, N. V., & Patel, D. (2023). Assessment of the plaque removal ability of two different dental floss devices: A comparative study. *World Journal of Dentistry, 4*(10), 860–863. <https://doi.org/10.5005/jp-journals-10015-2313>

Berger, S. A., Weitzman, S., Edberg, S. C., & Casey, J. I. (1974). Bacteraemia after the use of an oral irrigation device. A controlled study in subjects with normal appearing gingiva: Comparison with use of toothbrush. *Annals of Internal Medicine, 80*(4), 510–511. <https://doi.org/10.7326/0003-4819-80-4-510>

Bhaskar, S. N., Cutright, D. E., & Frisch, J. (1969). Effect of high pressure water jet on oral mucosa of varied density. *Journal of Periodontology, 40*(10), 593–598. <https://doi.org/10.1902/jop.1969.40.10.593>

Bhaskar, S. N., Cutright, D. E., Gross, A., Frisch, J., Beasley 3rd, J. D., & Perez, B. (1971). Water jet devices in dental practice. *Journal of Periodontology, 42*(10), 658–664. <https://doi.org/10.1902/jop.1971.42.10.658>

Bidra, A. S., Daubert, D. M., Garcia, L. T., Gauthier, M. F., Kosinski, T. F., Nenn, C. A., Olsen, J. A., Platt, J. A., Wingrove, S. S., Chandler, N. D., & Curtis, D. A. (2016). A systematic review of recall regimen and maintenance regimen of patients with dental restorations. Part 2: Implant-borne restorations. *Journal of Prosthodontics, 25*(S1), S16–S31. <https://doi.org/10.1111/jopr.12415>

Blumberg, E. J. (1973). Use of Water-Piks in acute chemical burns of the eye. *Texas Medicine, 69*(8), 92.

Bordabeheres, C. (2012). Water flosser and type 2 diabetes: Case report of bleeding and inflammation reduction. *RDH, 32*(4), 64–82.

Boyd, R. L., Hollander, B. N., & Eakle, W. S. (1992). Comparison of a subgingivally placed cannula oral irrigator tip with a supragingivally placed standard irrigator tip. *Journal of Clinical Periodontology, 9*(5), 340–344. <https://doi.org/10.1111/j.1600-051x.1992.tb00656.x>

Boyd, R. L., Leggott, P., Quinn, R., Buchanan, S., Eakle, W., & Chambers, D. (1985). Effect of self-administered daily irrigation with 0.02% SnF2 on periodontal disease activity. *Journal of Clinical Periodontology, 12*(6), 420–431. <https://doi.org/10.1111/j.1600-051X.1985.tb01378.x>

Brackett, M. G., Drisko, C. L., Thompson, A. L., Waller, J. L., Marshall, D. L., & Schuster, G. S. (2006). Penetration of fluids into periodontal pockets using a powered toothbrush/irrigator device. *Journal of Contemporary Dental Practice, 7*(3), 30–39.

Brady, J. M., Gray, W. A., & Bhaskar, S. N. (1973). Electron microscopic study of the effect of water lavage devices on dental plaque. *Journal of Dental Research, 52*(6), 1310–1313. <https://doi.org/10.1177/00220345730520062601>

Braun, R. E., & Ciancio, S. G. (1992). Subgingival delivery by an oral irrigation device. *Journal of Periodontology, 63*(5), 469–472. <https://doi.org/10.1902/jop.1992.63.5.469>

Brook, A. H., Duke, S., & Winter, G. B. (1977). Effect on plaque calcium and phosphorus concentration of short exposure to deionised water. *Caries Research, 11*(4), 243–244. <https://doi.org/10.1159/000260275>

Brownstein, C. N., Briggs, S. D., Schweitzer, K. L., Briner, W. W., & Kornman, K. S. (1990). Irrigation with chlorhexidine to resolve naturally occurring gingivitis. *Journal of Clinical Periodontology, 17*(8), 588–593. <https://doi.org/10.1111/j.1600-051x.1990.tb01110.x>

Bunk, D., Eisenburger, M., Häckl, S., Eberhard, J., Stiesch, M., & Grischke, J. (2020). The effect of adjuvant oral irrigation on self-administered oral care in the management of peri-implant mucositis: A randomized controlled clinical trial. *Clinical Oral Implants Research, 31*(10), 946–958. <https://doi.org/10.1111/clr.13638>

Burch, J. G., Lanese, R., & Ngan, P. (1994). A two-month study of the effects of oral irrigation and automatic toothbrush use in an adult orthodontic population with fixed appliances. *American Journal of Orthodontics and Dentofacial Orthopedics, 106*(2), 121–126. <https://doi.org/10.1016/S0889-5406(94)70028-1>

Butera, A., Gallo, S., Pascadopoli, M., Luraghi, G., & Scribante, A. (2021). Ozonized water administration in peri-implant mucositis sites: A randomized clinical trial. *Applied Sciences, 11*(17), Article 7812. <https://doi.org/10.3390/app11177812>

Caccianiga, P., Bader, A. A., Erba, P., & Caccianiga, G. (2022). Periodontal maintenance therapy: Efficacy of oral irrigator in the home oral hygiene protocol associated with microbiological analysis with phase contrast microscope. *Inventions, 7*(4), Article 104. <https://doi.org/10.3390/inventions7040104>

Caccianiga, P., Nota, A., Tecco, S., Ceraulo, S., & Caccianiga, G. (2022). Efficacy of home oral-hygiene protocols during orthodontic treatment with multibrackets and clear aligners: Microbiological analysis with phase-contrast microscope. *Healthcare, 10*(11), Article 2255. <https://doi.org/10.3390/healthcare10112255>

Cantor, M. T., & Stahl, S. S. (1969). Interdental col tissue responses to the use of a water pressure cleansing device. *Journal of Periodontology, 40*(5), 292–295. <https://doi.org/10.1902/jop.1969.40.5.292>

Carter, H. G., Barnes, G. P., Wooldridge, E. D., Jr., & Bhaskar, S. N. (1975). Evaluation of balsa wood stimulators and a water irrigation device on gingival sulcular bleeding. *Virginia Dental Journal, 52*(2), 17–29.

Chaves, E. S., Kornman, K. S., Manwell, M. A., Jones, A. A., Newbold, D. A., & Wood, R. C. (1994). Mechanism of irrigation effects on gingivitis. *Journal of Periodontology, 65*(11), 1016–1021. <https://doi.org/10.1902/jop.1994.65.11.1016>

Ciancio, S. G., Mather, M. L., Zambon, J. J., & Reynolds, H. S. (1989). Effect of a chemotherapeutic agent delivered by an oral irrigation device on plaque, gingivitis, and subgingival microflora. *Journal of Periodontology, 60*(6), 310–315. <https://doi.org/10.1902/jop.1989.60.6.310>

Clynes, J. T., & Wilderman, M. N. (1970). Effectiveness of a water-pressure device in removing debris from teeth. *Journal Of Public Health Dentistry, 30*(1), 2–6. <https://doi.org/10.1111/j.1752-7325.1970.tb00529.x>

Cobb, C. M., Rodgers, R. L., & Killoy, W. J. (1988). Ultrastructural examination of human periodontal pockets following the use of an oral irrigation device in vivo. *Journal of Periodontology, 59*(3), 155–163. <https://doi.org/10.1902/jop.1988.59.3.155>

Costa, F. O., Costa, A. A., & Cota, L. O. (2020). The use of interdental brushes or oral irrigators as adjuvants to conventional oral hygiene associated with recurrence of periodontitis in periodontal maintenance therapy: A 6-year prospective study. *Journal of Periodontology, 91*(1), 26–36. <https://doi.org/10.1002/JPER.18-0637>

Covin, N. R., Lainson, P. A., Belding, J. H., & Fraleigh, C. M. (1973). The effects of stimulating the gingiva by a pulsating water device. *Journal of Periodontology, 44*(5), 286–293. <https://doi.org/10.1902/jop.1973.44.5.286>

Crumley, P. J., & Sumner, C. F. (1965). Effectiveness of a water pressure cleaning device. *Periodontics, 3*(4), 193–195.

Cumming, B. R., & Löe, H. (1973). Optimal dosage and method of delivering chlorhexidine solutions for the inhibition of dental plaque. *Journal of Periodontal Research, 8*(2), 57–62. <https://doi.org/10.1111/j.1600-0765.1973.tb00751.x>

Curtis, J. P., Rustorgi, K. N., Petrone, M. E., & Volpe, A. R. (1988). Effect on gingival health of saline solution when applied as a mouth rinse or with a periodontal irrigating device [Abstract]. *Journal of Dental Research, 67*(Special issue), 400.

Cutler, C. W., Stanford, T. W., Abraham, C., Cederberg, R. A., Boardman, T. J., & Ross, C. (2000). Clinical benefits of oral irrigation for periodontitis are related to reduction of pro‐inflammatory cytokine levels and plaque. *Journal of Clinical Periodontology, 27*(2), 134–143. <https://doi.org/10.1034/j.1600-051x.2000.027002134.x>

Cutright, D. E., Beasley 3rd, J. D., Bhaskar, S. N., & Larson, W. J. (1973). Water lavage and tissue calibration study in rats. *Journal of Dental Research, 52*(1), 26–29. <https://doi.org/10.1177/00220345730520012901>

Cutright, D. E., Bhaskar, S. N., & Larson, W. J. (1972). Variable tissue forces produced by water jet devices. *Journal of Periodontology, 43*(12), 765–771. <https://doi.org/10.1902/jop.1972.43.12.765>

Deepa, A., Katuri, K. K., Swetha, C., Shivani, C. R., Boyapati, R., & Ravindranath, D. (2023). Clinical and microbiological efficacy of 0.25% sodium hypochlorite as a subgingival irrigant in chronic periodontitis patients: A pilot study. *World Journal of Dentistry, 14*(9), 745–750. <https://doi.org/10.5005/jp-journals-10015-2306>

Deepika, V., Chandrasekhar, R., Uloopi, K. S., Ratnaditya, A., Vinay, C., & RojaRamya, K. S. (2022). A randomized controlled trial for evaluation of the effectiveness of oral irrigator and interdental floss for plaque control in children with visual impairment. *International Journal of Clinical Pediatric Dentistry, 15*(4), 389–393. <https://doi.org/10.5005/jp-journals-10005-2404>

Derdivanis, J. P., Bushmaker, S., & Dagenais, F. (1978). Effects of a mouthwash in an irrigating device on accumulation and maturation of dental plaque. *Journal of Periodontology, 49*(2), 81–84. <https://doi.org/10.1902/jop.1978.49.2.81>

Dhingra, K., & Vandana, K. L. (2011). Management of gingival inflammation in orthodontic patients with ozonated water irrigation – A pilot study. *International Journal of Dental Hygiene, 9*(4), 296–302. <https://doi.org/10.1111/j.1601-5037.2011.00506.x>

Diekmann, J. M. (1984). Use of a dental irrigating device in the treatment of decubitus ulcers. *Nursing Research, 33*(5), 303–305.

Drapkin, M. S. (1977). Endocarditis after the use of an oral irrigation device. *Annals of Internal Medicine, 87*(4), 455. <https://doi.org/10.7326/0003-4819-87-4-455_1>

Drisko, C. L., White, C. L., Killoy, W. J., & Mayberry, W. E. (1987). Comparison of dark-field microscopy and a flagella stain for monitoring the effect of a Water Pik on bacterial motility. *Journal of Periodontology, 58*(6), 381–386. <https://doi.org/10.1902/jop.1987.58.6.381>

Dunkin, R. T. (1965). A new approach to oral physiotherapy with a new index of evaluation. *Journal of Periodontology, 36*(4), 315–321. <https://doi.org/10.1902/jop.1965.36.4.315>

Dunkin, R. T., Sumner 3rd, C. S., & Hughes, W. R. (1989a). An effectiveness study of a subgingival delivery system. *Quintessence International, 20*(5), 345–346.

Dunkin, R. T., Sumner 3rd, C. S., & Hughes, W. R. (1989b). Safety study of a subgingival delivery system. *Quintessence International, 20*(6), 401–402.

Duval, X., Millot, S., Chirouze, C., Selton-Suty, C., Moby, V., Tattevin, P., Strady, C., Euvrard, E., Agrinier, N., Thomas, D., Hoen, B., & Alla, F. (2017). Oral streptococcal endocarditis, oral hygiene habits, and recent dental procedures: A case-control study. *Clinical Infectious Diseases: An Official Publication of the Infectious Diseases Society of America, 64*(12), 1678–1685. <https://doi.org/10.1093/cid/cix237>

Eakle, W. S., Ford, C., & Boyd, R. L. (1986). Depth of penetration in periodontal pockets with oral irrigation. *Journal of Clinical Periodontology, 13*(1), 39–44. <https://doi.org/10.1111/j.1600-051x.1986.tb01412.x>

Eberhard, J., Damm, S., Freitag, S., Albers, H. K., & Jepsen, S. (2004). Plaque removing capacity of a novel high pressure water irrigator. *American Journal of Dentistry, 17*(3), 199–202.

Edlund, P., Bertl, K., Pandis, N., & Stavropoulos, A. (2023). Efficacy of power‐driven interdental cleaning tools: A systematic review and meta‐analysis. *Clinical and Experimental Dental Research, 9*(1), 3–16. <https://doi.org/10.1002/cre2.691>

Elliott, J. R., Bowers, G. M., Clemmer, B. A., & Rovelstad, G. H. (1972). A comparison of selected oral hygiene devices in dental plaque removal. *Journal of Periodontology, 43*(4), 217–220. <https://doi.org/10.1902/jop.1972.43.4.217>

ElShiekh, M. A., Alharbi, B. E., Alesayi, R. A., Albakri, R. A., & Alharbi, F. M. (2022). Prevalence of using dental water flosser during orthodontic treatment in Saudi Arabian population (Jeddah region). *International Journal of Advanced Research, 10*(2), 515–522. <https://doi.org/10.21474/IJAR01/14233>

Eltay, E. G., Gismalla, B. G., Mukhtar, M. M., & Awadelkarim, M. O. (2021). Punica granatum peel extract as adjunct irrigation to nonsurgical treatment of chronic gingivitis. *Complementary Therapies in Clinical Practice, 43*, Article 101383. <https://doi.org/10.1016/j.ctcp.2021.101383>

Ernst, C. P., Pittrof, M., Fürstenfelder, S., & Willershausen, B. (2004). Does professional preventive care benefit from additional subgingival irrigation? *Clinical Oral Investigations, 8*(4), 211–218. <https://doi.org/10.1007/s00784-004-0266-3>

Esposito, E. J., & Gray, W. A. (1975). Effect of water and mouthwashes on pH of oral monkey mucosa. *Pharmacology and Therapeutics in Dentistry, 2*(1), 33–41.

Fan, B., Ouyang, Z., Niu, J., Yu, S., & Rodrigues, J. (2018). Smart water flosser: A novel smart oral cleaner with IMU sensor. *IEEE Global Communications Conference (GLOBECOM)*, 1–7. <https://doi.org/10.1109/GLOCOM.2018.8647697>

Felix, J. E., Rosen, S., & App, G. R. (1971). Detection of bacteremia after the use of an oral irrigation device in subjects with periodontitis. *Journal of Periodontology, 42*(12), 785–787. <https://doi.org/10.1902/jop.1971.42.12.785>

Felo, A., Shibly, O., Ciancio, S. G., Lauciello, F. R., & Ho, A. (1997). Effects of subgingival chlorhexidine irrigation on peri-implant maintenance. *American Journal of Dentistry, 10*(2), 107–110.

Fine, D. H., & Baumhammers, A. (1970). Effect of water pressure irrigation on stainable material on the teeth. *Journal of Periodontology, 41*(8), 468–472. <https://doi.org/10.1902/jop.1970.41.8.468>

Fine, D. H., Korik, I., Furgang, D., Myers, R., Olshan, A., Barnett, M. L., & Vincent, J. (1996). Assessing pre-procedural subgingival irrigation and rinsing with an antiseptic mouthrinse to reduce bacteraemia. *Journal of the American Dental Association, 127*(5), 641–642. <https://doi.org/10.14219/jada.archive.1996.0276>

Fine, J. B., Harper, D. S., Gordon, J. M., Hovliaras, C. A., & Charles, C. H. (1994). Short-term microbiological and clinical effects of subgingival irrigation with an antimicrobial mouthrinse. *Journal of Periodontology, 65*(1), 30–36. <https://doi.org/10.1902/jop.1994.65.1.30>

Flemmig, T. F., Epp, B., Funkenhauser, Z., Newman, M. G., Kornman, K. S., Haubitz, I., & Klaiber, B. (1995). Adjunctive supragingival irrigation with acetylsalicylic acid in periodontal supportive therapy. *Journal of Clinical Periodontology, 22*(6), 427–433. <https://doi.org/10.1111/j.1600-051x.1995.tb00173.x>

Flemmig, T. F., Newman, M. G., Doherty, F. M., Grossman, E., Meckel, A. H., & Bakdash, M. B. (1990). Supragingival irrigation with 0.06% chlorhexidine in naturally occurring gingivitis. I. 6 month clinical observations. *Journal of Periodontology, 61*(2), 112–117. <https://doi.org/10.1902/jop.1990.61.2.112>

Flint, K. (2014). Water flossers preferred over string floss. *Perio Reports, 26*(11).

Frascella, J. A., Fernández, P., Gilbert, R. D., & Cugini, M. (2000). A randomized, clinical evaluation of the safety and efficacy of a novel oral irrigator. *American Journal of Dentistry, 13*(2), 55–58.

Ge, Y., Bamashmous, S., Mancinelli-Lyle, D., Zadeh, M., Mohamadzadeh, M., & Kotsakis, G. A. (2023). Interdental oral hygiene interventions elicit varying compositional microbiome changes in naturally occurring gingivitis: Secondary data analysis from a clinical trial. *Journal of Clinical Periodontology, 51*(3), 309–318. <https://doi.org/10.1111/jcpe.13899>

Gennai, S., Bollain, J., Ambrosio, N., Marruganti, C., Graziani, F., & Figuero, E. (2023). Efficacy of adjunctive measures in peri-implant mucositis. A systematic review and meta-analysis. *Journal of Clinical Periodontology, 50*(Suppl 26), 161–187. <https://doi.org/10.1111/jcpe.13791>

Genovesi, A. M., Lorenzi, C., Lyle, D. M., Marconcini, S., Barone, A., Pardo, A., Rigoni, G., Gelpi, F., Covani, U., & Bertossi, D. (2013). Periodontal maintenance following scaling and root planing. A randomized single-center study comparing minocycline treatment and daily oral irrigation with water. *Minerva Stomatologica, 62*(12), 1–9.

Gibbons, R. P., Correa Jr, R. J., Cummings, K. B., & Mason, J. T. (1974). Use of Water-Pik and nephroscope. *Urology, 4*(5), 605. <https://doi.org/10.1016/0090-4295(74)90504-4>

Gorur, A., Lyle, D. M., Schaudinn, C., & Costerton, J. W. (2009). Biofilm removal with a dental water jet. *Compendium of Continuing Education in Dentistry, 1*, 1–6.

Gosnell, N. E. (1981). *Toothbrushing, dental flossing and oral irrigation as devices in the prevention and treatment of dental disease* [Master's thesis, West Chester University of Pennsylvania]. ProQuest Dissertations & Theses Global. [www.proquest.com/dissertations-theses/toothbrushing-dental-flossing-oral-irrigation-as/docview/2218646808/se-2?accountid=8440https://resolver.ebscohost.com/openurl?ctx_ver=Z39.88-2004&ctx_enc=info:ofi/enc:UTF-8&rfr_id=info:sid/ProQuest+Dissertations+%26+Theses+Global&rft_val_fmt=info:ofi/fmt:kev:mtx:dissertation&rft.genre=dissertations+%26+theses&rft.jtitle=&rft.atitle=&rft.au=Gosnell%2C+Nancy+E.&rft.aulast=Gosnell&rft.aufirst=Nancy&rft.date=1981-01-01&rft.volume=&rft.issue=&rft.spage=&rft.isbn=978-1-392-03265-7&rft.btitle=&rft.title=Toothbrushing%2C+Dental+Flossing+and+Oral+Irrigation+as+Devices+in+the+Prevention+and+Treatment+of+Dental+Disease&rft.issn=&rft_id=info:doi/](file://C:\Users\fsark\Desktop\www.proquest.com\dissertations-theses\toothbrushing-dental-flossing-oral-irrigation-as\docview\2218646808\se-2?accountid=8440https:\\resolver.ebscohost.com\openurl?ctx_ver=Z39.88-2004&ctx_enc=info:ofi\enc:UTF-8&rfr_id=info:sid\ProQuest+Dissertations+&+Theses+Global&rft_val_fmt=info:ofi\fmt:kev:mtx:dissertation&rft.genre=dissertations+&+theses&rft.jtitle=&rft.atitle=&rft.au=Gosnell,+Nancy+E.&rft.aulast=Gosnell&rft.aufirst=Nancy&rft.date=1981-01-01&rft.volume=&rft.issue=&rft.spage=&rft.isbn=978-1-392-03265-7&rft.btitle=&rft.title=Toothbrushing,+Dental+Flossing+and+Oral+Irrigation+as+Devices+in+the+Prevention+and+Treatment+of+Dental+Disease&rft.issn=&rft_id=info:doi\)

Goyal, C. R., Lyle, D. M., Qaqish, J. G., & Schuller, R. (2012). The addition of a water flosser to power tooth brushing: Effect on bleeding, gingivitis, and plaque. *Journal of Clinical Dentistry, 23*(2), 57–63.

Goyal, C. R., Lyle, D. M., Qaqish, J. G., & Schuller, R. (2013). Evaluation of the plaque removal efficacy of a water flosser compared to string floss in adults after a single use. *Journal of Clinical Dentistry, 24*(2), 37–42.

Goyal, C. R., Lyle, D. M., Qaqish, J. G., & Schuller, R. (2015). Efficacy of two interdental cleaning devices on clinical signs of inflammation: A four-week randomized controlled trial. *Journal of Clinical Dentistry, 26*(2), 55–60.

Goyal, C. R., Lyle, D. M., Qaqish, J. G., & Schuller, R. (2016). Comparison of water flosser and interdental brush on reduction of gingival bleeding and plaque: A randomized controlled pilot study. *The Journal of Clinical Dentistry, 27*(2), 61–65.

Goyal, C. R., Qaqish, J. G., Schuller, R., & Lyle, D. M. (2018a). Comparison of a novel sonic toothbrush with a traditional sonic toothbrush and manual brushing and flossing on plaque, gingival bleeding, and inflammation: A randomized controlled clinical trial. *Compendium of Continuing Education in Dentistry, 39*(2), 14–20.

Goyal, C. R., Qaqish, J. G., Schuller, R., & Lyle, D. M. (2018b). Direct comparison of a novel sonic toothbrush with a traditional sonic toothbrush on clinical signs of inflammation: A randomized controlled pilot study. *Compendium of Continuing Education in Dentistry, 39*(2), 6–7.

Goyal, C. R., Qaqish, J. G., Schuller, R., & Lyle, D. M. (2018c). Evaluation of the addition of a water flosser to manual brushing on gingival health. *Journal of Clinical Dentistry, 29*(4), 81–86.

Goyal, C. R., Qaqish, J. G., Schuller, R., & Lyle, D. M. (2018d). Evaluation of the safety of a water flosser on gingival and epithelial tissue at different pressure settings. *Compendium of Continuing Education in Dentistry, 39*(2), 8–13.

Gross, A., Bhaskar, S. N., Cutright, D. E., Beasley, J. D., & Perez, B. (1971). The effect of pulsating water jet lavage on experimental contaminated wounds. *Journal of Oral Surgery, 29*(3), 187–190.

Grossan, M. (1974). Irrigation of the child's nose: Successful application of a dental pulsating irrigation device. *Clinical Pediatrics, 13*(3), 229–231. <https://doi.org/10.1177/000992287401300306>

Grower, M. F., & Bhaskar, S. N. (1972). Effect of pulsating water jet lavage on radioactive contaminated wounds. *Journal of Dental Research, 51*(2), 536–538. <https://doi.org/10.1177/00220345720510024801>

Gupta, O. P., O'Toole, E. T., & Hammermeister, R. O. (1973). Effects of a water pressure device on oral hygiene and gingival inflammation. *Journal of Periodontology, 44*(5), 294–298. <https://doi.org/10.1902/jop.1973.44.5.294>

Hemalatha, S., Srinivasan, A., Srirekha, A., Santhosh, L., Champa, C., & Shetty, A. (2022). An in vitro radiological evaluation of irrigant penetration in the root canals using three different irrigation systems: Waterpik WP‑100 device, passive irrigation, and manual dynamic irrigation systems. *Journal of Conservative Dentistry, 25*(4), 403–408. <https://doi.org/10.4103/jcd.jcd_162_22>

Hentenaar, D. F., De Waal, Y. C., Van Winkelhoff, A. J., Meijer, H. J., & Raghoebar, G. (2020). Non-surgical peri-implantitis treatment using a pocket irrigator device; clinical, microbiological, radiographical and patient-centred outcomes—A pilot study. *International Journal of Dental Hygiene, 18*(4), 403–412. <https://doi.org/10.1111/idh.12462>

Herzog, A., & Hodges, K. O. (1988). Subgingival irrigation with Chloramine-T. *Journal of Dental Hygiene, 62*(10), 515–521.

Hock, J., & Tinanoff, N. (1979). Resolution of gingivitis in dogs following topical applications of 0.4% stannous fluoride and toothbrushing. *Journal of Dental Research, 58*(6), 1652–1653. <https://doi.org/10.1177/00220345790580061601>

Hoexter, B. (1976). Use of Water Pik lavage in pilonidal wound care. *Diseases of the Colon and Rectum, 19*(5), 470–471. <https://doi.org/10.1007/BF02590836>

Hoover, D. R., & Robinson, H. B. (1971). The comparative effectiveness of a pulsating oral irrigator as an adjunct in maintaining oral health. *Journal of Periodontology, 42*(1), 37–39. <https://doi.org/10.1902/jop.1971.42.1.37>

Hoover, D. R., Robinson, H. B., & Billingsley, A. (1968). The comparative effectiveness of the Water-Pik in a noninstructed population [Abstract]. *Journal of Periodontology, 39*(1), 43.

Hugoson, A. (1978). Effect of the Water Pik® device on plaque accumulation and development of gingivitis. *Journal of Clinical Periodontology, 5*(2), 95–104. <https://doi.org/10.1111/j.1600-051x.1978.tb01911.x>

Hurst, J. E., & Madonia, J. V. (1970). The effect of an oral irrigating device on the oral hygiene of orthodontic patients. *Journal of the American Dental Association, 81*(3), 678–682. <https://doi.org/10.14219/jada.archive.1970.0313>

Husseini, A., Slot, D. E., & Van der Weijden, G. A. (2008). The efficacy of oral irrigation in addition to a toothbrush on plaque and the clinical parameters of periodontal inflammation: A systematic review. *International Journal of Dental Hygiene, 6*(4), 304–314. <https://doi.org/10.1111/j.1601-5037.2008.00343.x>

Hygienetown. (2010, May). Hygienists' opinions about oral irrigation. *Hygienetown*, *6*(4), 12.

Ioannidis, A., Thurnheer, T., Hofer, D., Sahrmann, P., Guggenheim, B., & Schmidlin, P. R. (2015). Mechanical and hydrodynamic homecare devices to clean rough implant surfaces – An in vitro polyspecies biofilm study. *Clinical Oral Implants Research, 26*(5), 523–528. <https://doi.org/10.1111/clr.12436>

Issac, A. V., Mathew, J. J., Ambooken, M., Kachappilly, A. J., Ajithkumar, P. K., Johny, T., Linith, V. K., & Samuel, A. (2015). Management of chronic periodontitis using subgingival irrigation of ozonized water: A clinical and microbiological study. *Journal of Clinical and Diagnostic Research, 9*(8), ZC29–ZC33. <https://doi.org/10.7860/JCDR/2015/14464.6303>

Isshiki, Y. (1970). Effect of oral cleansing in cerebral palsied children (application of a water jet device). *The Bulletin of Tokyo Dental College, 11*(2), 121–131.

Itic, J., & Serfaty, R. (1992). Clinical effectiveness of subgingival irrigation with a pulsated jet irrigator versus syringe. *Journal of Periodontology, 63*(3), 174–181. <https://doi.org/10.1902/jop.1992.63.3.174>

Jackson, C. L. (1991). Comparison between electric toothbrushing and manual toothbrushing, with and without oral irrigation, for oral hygiene of orthodontic patients. *American Journal of Orthodontics and Dentofacial Orthopedics, 99*(1), 15–20. <https://doi.org/10.1016/S0889-5406(05)81675-0>

Jain, R., Chaturvedi, R., Pandit, N., Grover, V., Lyle, D. M., & Jain, A. (2020). Evaluation of the efficacy of subgingival irrigation in patients with moderate-to-severe chronic periodontitis otherwise indicated for periodontal flap surgeries. *Journal of Indian Society of Periodontology, 24*(4), 348–353. <https://doi.org/10.4103/jisp.jisp_54_20>

Jalaluddin, M., Mailankote, S., Sam, G., Penumatsa, N. V., Alazmah, A., & Punde, P. (2020). Assessment of the efficacy of various subgingival irrigating solutions in chronic periodontitis: A comparative study. *World Journal of Dentistry, 11*(3), 221–225. <https://doi.org/10.5005/jp-journals-10015-1735>

Johnson, K. E., Sanders, J. J., Gellin, R. G., & Palesch, Y. Y. (1998). The effectiveness of a magnetized water orai irrigator (Hydro Fioss®) on plaque, calculus and gingival health. *Journal of Clinical Periodontology, 25*(4), 316–321. <https://doi.org/10.1111/j.1600-051x.1998.tb02447.x>

Jolkovsky, D. L., Waki, M. Y., Newman, M. G., Otomo-Corgel, J., Madison, M., Flemmig, T. F., Nachnani, S., & Nowzari, H. (1990). Clinical and microbiological effects of subgingival and gingival marginal irrigation with chlorhexidine gluconate. *Journal of Periodontology, 61*(11), 663–669. <https://doi.org/10.1902/jop.1990.61.11.663>

Jones, C. M., Blinkhorn, A. S., & White, E. (1990). Hydrogen peroxide, the effect on plaque and gingivitis when used in an oral irrigator. *Clinical Preventive Dentistry, 12*(5), 15–18.

Jose, P., Ramabhadran, B. K., Emmatty, R., & Paul, T. P. (2017). Assessment of the effect of ozonated water irrigation on gingival inflammation in patients undergoing fixed orthodontic treatment. *Journal of Indian Society of Periodontology, 21*(6), 484–488. <https://doi.org/10.4103/jisp.jisp_265_16>

Kancir, S. L., & Krajewski, J. J. (1972). The relation of water pressure cleansing to the reticulo-endothelial system. *Journal of Periodontology, 43*(11), 696–698. <https://doi.org/10.1902/jop.1972.43.11.696>

Kaplan, E. L., & Anderson, R. C. (1977). Infective endocarditis after use of dental irrigation device. *Lancet, 2*(8038), 610. <https://doi.org/10.1016/s0140-6736(77)91460-x>

Kato, K., Tamura, K., & Nakagaki, H. (2012). Quantitative evaluation of the oral biofilm-removing capacity of a dental water jet using an electron-probe microanalyzer. *Archives of Oral Biology, 57*(1), 30–35. <https://doi.org/10.1016/j.archoralbio.2011.08.006>

Katti, S. S., & Chava, V. K. (2013). Effect of ozonised water on chronic periodontitis – A clinical study. *Journal of International Oral Health, 5*(5), 79–84.

Katz, S., Katzka, I., Platt, N., Hajdu, E. O., & Bassett, E. (1977). Cancer in chronic ulcerative colitis. Diagnostic role of segmental colonic lavage. *The American Journal of Digestive Diseases, 22*(4), 355–364. <https://doi.org/10.1007/BF01072194>

Kaur, A., Bhavikatti, S. K., Das, S. S., Khanna, S., Jain, M., & Kaur, A. (2019). Efficacy of ozonised water and 0.2% chlorhexidine gluconate in the management of chronic periodontitis when used as an irrigant in conjugation with phase I therapy. *Journal of Contemporary Dental Practice, 20*(3), 318–323.

Kaur, J., Grover, V., Gupta, J., Gupta, M., Kapur, V., Mehta, M., Lyle, D. M., Samujh, T., & Jain, A. (2023). Effectiveness of subgingival irrigation and powered toothbrush as home care maintenance protocol in type 2 diabetic patients with active periodontal disease: A 4-month randomized controlled trial. *Journal of Indian Society of Periodontology, 27*(5), 515–523. <https://doi.org/10.4103/jisp.jisp_509_21>

Kelly, A., Resteghini, R., Williams, B., & Dolby, A. E. (1985). Pressures recorded during periodontal pocket irrigation. *Journal of Periodontology, 56*(5), 297–299. <https://doi.org/10.1902/jop.1985.56.5.297>

Kesavan, S. K., Reddy, N. P., & Yazdani-Ardakani, S. (1986). Experimental measurement of impact pressures delivered by oral water irrigation devices. *IEEE Transactions on Bio-Medical Engineering, 33*(9), 898–900. <https://doi.org/10.1109/TBME.1986.325788>

Khaimov, A., & Tobias, G. (2020). Efficacy of ozonated water adjunct to regular oral hygiene maintenance in moderate gingivitis: A randomized clinical trial. *Open Access Journal of Dental Sciences, 5*(5), Article 000269. <https://doi.org/10.23880/oajds-16000269>

Khatri, M., Malik, A. S., Bansal, M., Puri, K., Gupta, G., & Kumar, A. (2017). Effect of supragingival oral irrigation as an adjunct to toothbrushing on plaque accumulation in chronic generalized gingivitis patients. *Journal of Indian Society of Periodontology, 21*(4), 296–302. <https://doi.org/10.4103/jisp.jisp_393_15>

Kim, J. M., Yoo, S. Y., An, J. S., Woo, J. J., Cho, Y. D., Park, H. E., & Karm, M. H. (2023). Effect of a multichannel oral irrigator on periodontal health and the oral microbiome. *Scientific Reports, 13*(1), Article 12043. <https://doi.org/10.1038/s41598-023-38894-0>

Kim, J. Y., Bae, E. B., Sung, E. C., Lee, T., Camargo, P., Shin, K. H., Kim, H. J., Pyun, W. B., & Kim, R. H. (2023). Effects of the multi-channeled oral irrigation (MCOI) unit in preventing dental plaque formation and gingivitis: A randomized controlled trial. *American Journal of Dentistry, 36*(5), 215–221.

Korn, E. R. (1972). Use of a dental irrigating unit in the treatment of fecal impactions. *Gastrointestinal Endoscopy, 19*(2), 88. <https://doi.org/10.1016/s0016-5107(72)73966-8>

Kotsakis, G. A., Black, R., Kum, J., Berbel, L., Sadr, A., Karoussis, I., Simopoulou, M., & Daubert, D. (2021). Effect of implant cleaning on titanium particle dissolution and cytocompatibility. *Journal of Periodontology, 92*(4), 580–591. <https://doi.org/10.1002/JPER.20-0186>

Kotsakis, G. A., Lian, Q., Ioannou, A. L., Michalowicz, B. S., John, M. T., & Chu, H. A. (2018). A network meta-analysis of interproximal oral hygiene methods in the reduction of clinical indices of inflammation. *Journal of Periodontology, 89*(5), 558–570. <https://doi.org/10.1002/JPER.17-0368>

Kozam, G. (1973). The effect of hydromassage on capillary strength. *The New York State Dental Journal, 39*(9), 551–559.

Krajewski, J., Giblink, J., & Gargiulo, A. (1964). Evaluation of a water pressure cleaning device as an adjunct to periodontal treatment. *Periodontics, 2*(2), 76–78.

Krajewski, J. J., Rubach, W. C., & Pope, J. W. (1967). The effect of water pressure cleansing on the clinically normal gingival crevice. *Journal of the California Dental Association, 43*(5), 452–454.

Krishna, M. K., Ravindran, S. K., Vivekanandan, G., Navasivayam, A., Thiagarajan, R., & Mohan, R. (2011). Effects of a single episode of subgingival irrigation with tetracycline HCl or chlorhexidine: A clinical and microbiological study. *Journal of Indian Society of Periodontology, 15*(3), 245–249. <https://doi.org/10.4103/0972-124X.85668>

Krugman, M. E., & Beumer, J. (1975). Maxillectomy cavity care with a pulsating stream irrigator. *Eye, Ear, Nose & Throat Monthly, 54*(3), 104.

Kshitish, D., & Laxman, V. K. (2010). The use of ozonated water and 0.2% chlorhexidine in the treatment of periodontitis patients: A clinical and microbiologic study. *Indian Journal of Dental Research, 21*(3), 341–348. <https://doi.org/10.4103/0970-9290.70796>

Lainson, P. A., Bergquist, J. J., & Fraleigh, C. M. (1970). Clinical evaluation of Pulsar, a new pulsating water pressure cleansing device. *Journal of Periodontology, 41*(7), 401–405. <https://doi.org/10.1902/jop.1970.41.7.401>

Lainson, P. A., Bergquist, J. J., & Fraleigh, C. M. (1972). A longitudinal study of pulsating water pressure cleansing devices. *Journal of Periodontology, 43*(7), 444–446. <https://doi.org/10.1902/jop.1972.43.7.444>

Lainson, P. A., Bergquist, J. J., Tade, W. H., & Fraleigh, C. M. (1971). A histopathological study of tissue responses to the pulsar pulsating water pressure cleansing device. *Journal of Periodontology, 42*(2), 101–104. <https://doi.org/10.1902/jop.1971.42.2.101>

Lang, N. P., & Ramseier-Grossmann, K. (1981). Optimal dosage of chlorhexidine digluconate in chemical plaque control when applied by the oral irrigator. *Journal of Clinical Periodontology, 8*(3), 189–202. <https://doi.org/10.1111/j.1600-051X.1981.tb02030.x>

Lang, N. V., & Räber, K. (1981). Use of oral irrigators as vehicle for the application of antimicrobial agents in chemical plaque control. *Journal of Clinical Periodontology, 8*(3), 177–188. <https://doi.org/10.1111/j.1600-051X.1981.tb02029.x>

Larner, J. R., & Greenstein, G. (1993). Effect of calculus and irrigator tip design on depth of subgingival irrigation. *International Journal of Periodontics and Restorative Dentistry, 13*(3), 288–297.

Levin, L., Frankenthal, S., Joseph, L., Rozitsky, D., Levi, G., & Machtei, E. E. (2015). Water jet with adjunct chlorhexidine gel for nonsurgical treatment of peri-implantitis. *Quintessence International, 46*(2), 133–137. <https://doi.org/10.3290/j.qi.a32819>

Lin, P. J., Chuang, M. C., & Chang, S. C. (2018). Efficacy of using oxygen microbubble device for facultative anaerobe removal. *IET NBT Nanobiotechnology, 12*(7), 973–980. <https://doi.org/10.1049/iet-nbt.2017.0232>

Listgarten, M. A., Grossberg, D., Schwimer, C., Vito, A., & Gaffar, A. (1989). Effect of subgingival irrigation with tetrapotassium peroxydiphosphate on scaled and untreated periodontal pockets. *Journal of Periodontology, 60*(1), 4–11. <https://doi.org/10.1902/jop.1989.60.1.4>

Lobene, R. R. (1969). The effect of a pulsed water pressure cleansing device on oral health. *Journal of Periodontology, 40*(1), 667–670. <https://doi.org/10.1902/jop.1969.40.11.667>

Lobene, R. R. (1971). A study of the force of water jets in relation to pain and damage to gingival tissues. *Journal of Periodontology, 42*(3), 166–169. <https://doi.org/10.1902/jop.1971.42.3.166>

Lobene, R. R., Soparkar, P. M., Hein, J. W., & Quigley, G. A. (1972). A study of the effects of antiseptic agents and a pulsating irrigating device on plaque and gingivitis. *Journal of Periodontology, 43*(9), 564–568. <https://doi.org/10.1902/jop.1972.43.9.564>

Lofthus, J. E., Waki, M. Y., Jolkovsky, D. L., Otomo-Corgel, J., Newman, M. G., Flemmig, T., & Nachnani, S. (1991). Bacteraemia following subgingival irrigation and scaling and root planing. *Journal of Periodontology, 62*(10), 602–607. <https://doi.org/10.1902/jop.1991.62.10.602>

Logan, E. I. (1994). *A model for evaluation of supragingival plaque and the effects of mechanical and chemical plaque control in the dog* [Doctoral thesis, Kansas State University]. ProQuest Dissertations and Theses Global. <https://www.proquest.com/openview/f786658e75034bfb7ef22c1c557ca9bd/1?pq-origsite=gscholar&cbl=18750&diss=y>

Loud, B. (2001). A WaterPik to clear sinuses? *RN, 64*(1), 48–49.

Lugassy, A. A., & Lautenschlager, E. P. (1970). Water spray cleansing action in oral hygiene. *The Journal of the American Dental Hygienists' Association, 45*(4), 51–53.

Lugassy, A. A., Lautenschlager, E. P., & Katrana, D. (1971). Characterization of water spray devices. *Journal of Dental Research, 50*(2), 466–473. <https://doi.org/10.1177/00220345710500025901>

Luo, Y., Wang, D., Li, Y., Geng, D., Yu, B., Zhao, Y., & Xiang, Q. (2023). Digital imaging and qPCR analysis and comparison of short-term plaque removal effects of tooth brushing. *Frontiers in Dental Medicine, 4*, Article 1103602. <https://doi.org/10.3389/fdmed.2023.1103602>

Lyle, D. M., Goyal, C. R., Qaqish, J. G., & Schuller, R. (2016). Comparison of water flosser and interdental brush on plaque removal: A single-use pilot study. *The Journal of Clinical Dentistry, 27*(1), 23–26.

Lyle, D. M., Qaqish, J. G., Goyal, C. R., & Schuller, R. (2020). Efficacy of the use of a water flosser in addition to an electric toothbrush on clinical signs of inflammation: 4-week randomized controlled trial. *Compendium of Continuing Education in Dentistry, 41*(3), 170–177.

Macaulay, W. J., & Newman, H. N. (1986). The effect on the composition of subgingival plaque of a simplified oral hygiene system including pulsating jet subgingival irrigation. *Journal of Periodontal Research, 21*(4), 375–385. <https://doi.org/10.1111/j.1600-0765.1986.tb01471.x>

Magnuson, B., Harsono, M., Stark, P. C., Lyle, D., Kugel, G., & Perry, R. (2013). Comparison of the effect of two interdental cleaning devices around implants on the reduction of bleeding: a 30-day randomized clinical trial. *Compendium of Continuing Education in Dentistry, 34*(8), 2–7.

Mancinelli-Lyle, D., Qaqish, J. G., Goyal, C. R., & Schuller, R. (2023a). Effectiveness of a sonic-flosser toothbrush with different size brush heads and manual toothbrush plus dental floss on plaque, gingival bleeding and inflammation in adults with naturally occurring gingivitis: A 4-week randomized controlled trial. *International Journal of Dental Hygiene, 21*(3), 624–633. <https://doi.org/10.1111/idh.12675>

Mancinelli-Lyle, D., Qaqish, J. G., Goyal, C. R., & Schuller, R. (2023b). Efficacy of water flossing on clinical parameters of inflammation and plaque: A four-week randomized controlled trial. *International Journal of Dental Hygiene, 21*(4), 659–668. <https://doi.org/10.1111/idh.12770>

Manhold, J. H., & Knutsen, M. (1992). A retrospective of normal oral healthcare procedures vs. power brush/jet lavage with a corroborative study. *Clinical Preventive Dentistry, 14*(4), 10–15.

Manhold, J. H., Vogel, R. I., & Manhold, E. A. (1978). Carbon penetration of gingival tissue by oral irrigating devices. *The Journal of Preventive Dentistry, 5*(5), 3–6.

Matthes, R., Jablonowski, L., Miebach, L., Pitchika, V., Holtfreter, B., Eberhard, C., Seifert, L., Gerling, T., Schlüter, R., Kocher, T., & Bekeschus, S. (2023). In-vitro biofilm removal efficacy using water jet in combination with cold plasma technology on dental titanium implants. *International Journal of Molecular Sciences, 24*(2), Article 1606. <https://doi.org/10.3390/ijms24021606>

Matthes, R., Jablonowski, L., Pitchika, V., Holtfreter, B., Eberhard, C., Seifert, L., Gerling, T., Vilardell Scholten, L., Schlüter, R., & Kocher, T. (2022). Efficiency of biofilm removal by combination of water jet and cold plasma: An in-vitro study. *BMC Oral Health, 22*(1), Article 157. <https://doi.org/10.1186/s12903-022-02195-1>

McDevitt, M. J., & Eames, W. B. (1971). Attrition of dental restorations by a pulsating water device. *Virginia Dental Journal, 48*(1), 6–10.

Meklas, F., & Stewart, J. L. (1972). Investigation of the safety and effectiveness of an oral irrigating device. *Journal of Periodontology, 43*(7), 441–443. <https://doi.org/10.1902/jop.1972.43.7.441>

Moore, G. C., Smith, K. T., Christiansen, M. M., Anderson, L., Moravec, L. J., Okano, D. K., Samson, K. K., Ramer-Tait, A., Beede, K., Reinhardt, R. A., & Killeen, A. C. (2023). Effect of interproximal home oral hygiene on clinical parameters and inflammatory biomarkers in patients receiving periodontal maintenance. *Journal of Periodontology, 94*(7), 848–857. <https://doi.org/10.1002/JPER.22-0631>

Murthy, P. S., Shaik, N., Deshmukh, S., & Girish, M. S. (2018). Effectiveness of plaque control with novel pediatric oral hygiene need station (modified oral irrigation device) as compared with manual brushing and flossing: Randomized controlled pilot trial. *Contemporary Clinical Dentistry, 9*(2), 170–173. <https://doi.org/10.4103/ccd.ccd_749_17>

Naser-Alavi, F., Salari, A., Moein, N., & Talebzadeh, A. (2022). Effect of oral irrigation device and its solution type on the surface roughness and topography of bulk-fill composite resins. *Journal of Clinical and Experimental Dentistry, 14*(2), e123–e130. <https://doi.org/10.4317/jced.59004>

Newman, M. G., Cattabriga, M., Etienne, D., Flemmig, T., Sanz, M., Kornman, K. S., Doherty, F., Moore, D. J., & Ross, C. (1994). Effectiveness of adjunctive irrigation in early periodontitis: Multi-center evaluation. *Journal of Periodontology, 65*(3), 224–229. <https://doi.org/10.1902/jop.1994.65.3.224>

Newman, M. G., Flemmig, T. F., Nachnani, S., Rodrigues, A., Calsina, G., Lee, Y. S., de Camargo, P., Doherty, F. M., & Bakdash, M. B. (1990). Irrigation with 0.06% chlorhexidine in naturally occurring gingivitis. II. 6 months microbiological observations. *Journal of Periodontology, 61*(7), 427–433. <https://doi.org/10.1902/jop.1990.61.7.427>

Nie, J. (2017). *Oral hygiene system design* [Master's thesis, Rochester Institute of Technology]. RIT Scholar Works. <https://scholarworks.rit.edu/theses/9436/>

O'Leary, T. J., Shafer, W. G., Swenson, H. M., Nesler, D. C., & Van Dorn, P. R. (1970). Possible penetration of crevicular tissue from oral hygiene procedures. I. Use of oral irrigating devices. *Journal of Periodontology, 41*(3), 158–162. <https://doi.org/10.1902/jop.1970.41.41.158>

Oshrain, R. L., Fiorello, L. A., Harper, D. S., & Lamster, I. B. (1987). Oral irrigation devices. A clinical evaluation. *Dental Hygiene, 61*(12), 551–555.

Pandya, D. J., Manohar, B., Mathur, L. K., & Shankarapillai, R. (2016). Comparative evaluation of two subgingival irrigating solutions in the management of periodontal disease: A clinicomicrobial study. *Journal of Indian Society of Periodontology, 20*(6), 597–602. <https://doi.org/10.4103/jisp.jisp_328_16>

Park, S. Y., Kim, K. H., Shin, S. Y., Koo, K. T., Lee, Y. M., Chung, C. P., & Seol, Y. J. (2015). Decontamination methods using a dental water jet and dental floss for microthreaded implant fixtures in regenerative periimplantitis treatment. *Implant Dentistry, 24*(3), 307–316. <https://doi.org/10.1097/ID.0000000000000208>

Parsons, L. G., Thomas, L. G., Southard, G. L., Woodall, I. R., & Jones, B. J. (1987). Effect of sanguinaria extract on established plaque and gingivitis when supragingivally delivered as a manual rinse or under pressure in an oral irrigator. *Journal of Clinical Periodontology, 14*(7), 381–385. <https://doi.org/10.1111/j.1600-051X.1987.tb01540.x>

Parsons, L. G., Thomas, L. G., Woodall, I. R., & Jones, B. J. (1986). Effect of 0.03% sanguinaria rinse on plaque and gingivitis when delivered as a manual rinse and under pressure in an oral irrigator. *Compendium of Continuing Education in Dentistry, S7*, S205–S208.

Patel, D., Mehta, F., Trivedi, I., Mehta, N., Shah, U., & Vaghela, V. (2015). Study of the effects of oral irrigation and automatic tooth brush use in orthodontic patients with fixed appliances. *Journal of Orthodontic Research, 3*(1), 4–10. <https://doi.org/10.4103/2321-3825.147973>

Perayil, J., Menon, K. S., Biswas, R., Fenol, A., & Vyloppillil, R. (2016). Comparison of the efficacy of subgingival irrigation with 2% povidone‑iodine and tetracycline HCl in subjects with chronic moderate periodontitis: A clinico-microbiological study. *Dental Research Journal, 13*(2), 98–109.

Peterson, W. A., & Shiller, W. R. (1968). Unsupervised use of a water spray device by naval personnel. *Journal of Periodontology, 39*(6), 335–337. <https://doi.org/10.1902/jop.1968.39.6.335>

Phelps-Sandall, B. A., & Oxford, S. J. (1983). Effectiveness of oral hygiene techniques on plaque and gingivitis in patients placed in intermaxillary fixation. *Oral surgery, oral medicine, and oral pathology, 56*(5), 487–490. <https://doi.org/10.1016/0030-4220(83)90094-4>

Pistorius, A., Willershausen, B., Steinmeier, E. M., & Kreislert, M. (2003). Efficacy of subgingival irrigation using herbal extracts on gingival inflammation. *Journal of Periodontology, 74*(5), 616–622. <https://doi.org/10.1902/jop.2003.74.5.616>

Pithon, M. M., Sant'Anna, L. I., Baião, F. C., Coqueiro, R. S., Maia, L. C., & Paranhos, L. R. (2017). Effectiveness of different mechanical bacterial plaque removal methods in patients with fixed orthodontic appliance: A systematic review and meta-analysis. *Bioscience Journal, 33*(2), 537–554. <https://doi.org/10.14393/BJ-v33n2-35755>

Rams, T. E., & Keyes, P. H. (1984). Modifying oral irrigation devices for subgingival periodontal chemotherapy. *General Dentistry, 32*(4), 302–305.

Rams, T. E., & Slots, J. (2023). Antimicrobial chemotherapy for recalcitrant severe human periodontitis. *Antibiotics, 12*(2), Article 265. <https://doi.org/10.3390/antibiotics12020265>

Ramseier, C. A., Petitat, C., Trepp, S., Lang, N. P., Eick, S., Adam, R., Ccahuana-Vasquez, R. A., Barker, M. L., Timm, H., Klukowska, M., & Salvi, G. E. (2021). Clinical parameters and oral fluid biomarkers in gingivitis subjects using an electric toothbrush with Irrigator vs a manual toothbrush alone over 8 Weeks: A randomised controlled clinical trial. *Oral Health & Preventive Dentistry, 19*(1), 137–148. <https://doi.org/10.3290/j.ohpd.b966767>

Ravindrareddy, N., Madhubabu, D. S., Mahendraraju, C., Himasilpa, C., & Kiran, K. (2012). Clinical effects of professional antimicrobial subgingival pulsated jet irrigation [WaterPik®] in patients with chronic periodontitis: A comparative study. *Journal of Dr. NTR University of Health Sciences, 1*(1), 38–45. <https://doi.org/10.4103/2277-8632.94174>

Ravishankar, P. L., Venugopal, K., & Nadkerny, P. (2015). Effect of tetracycline hydrochloride and spiramycin subgingival irrigation with pulsated jet irrigator in chronic periodontitis patients: A clinical study. *Journal of International Oral Health, 7*(7), 102–107.

Reddy, N. P., Kesavan, S. K., & Costarella, B. R. (1985). Evaluation of oral water irrigation devices. *Engineering in Medicine, 14*(3), 141–145. <https://doi.org/10.1243/emed_jour_1985_014_033_02>

Ren, X., He, J., Cheng, R., Chen, Y., Xiang, Y., Zhang, Y., Jiang, S., Li, J., Cheng, L., & Hu, T. (2023). The efficacy and safety of oral irrigator on the control of dental plaque and gingivitis: A randomized, single-blind, parallel-group clinical trial. *International Journal of Environmental Research and Public Health, 20*(4), Article 3726. <https://doi.org/10.3390/ijerph20043726>

Romans, A. R., & App, G. R. (1971). Bacteraemia, a result from oral irrigation in subjects with gingivitis. *Journal of Periodontology, 42*(12), 757–760. <https://doi.org/10.1902/jop.1971.42.12.757>

Rose, J., Ghoneima, A., Lippert, F., Maxwell, L., Eckert, G. J., & Stewart, K. T. (2020). A visual evaluation of oral plaque removal utilizing an adjunct enzyme pre-rinse in orthodontic subjects. *Angle Orthodontist, 90*(6), 844–850. <https://doi.org/10.2319/120819-776.1>

Rosema, N. A. M., Hennequin-Hoenderdos, N. L., Berchier, C. E., Slot, D. E., Lyle, D. M., & van der Weijden, G. A. (2011). The effect of different interdental cleaning devices on gingival bleeding. *Journal of the International Academy of Periodontology, 13*(1), 2–10.

Rüdiger, S., Petersilka, G., & Flemmig, T. F. (1999). Combined systemic and local antimicrobial therapy of periodontal disease in Papillon-Lefèvre syndrome. A report of 4 cases. *Journal of Clinical Periodontology, 26*(12), 847–854.

Salles, M. M., de Cássia Oliveira, V., Macedo, A. P., Silva-Lovato, C., & de Freitas de Oliveira Paranhos, H. (2021). Effectiveness of brushing associated with oral irrigation in maintenance of peri-implant tissues and overdentures: Clinical parameters and patient satisfaction. *The Journal of Oral Implantology, 47*(2), 117–123. <https://doi.org/10.1563/aaid-joi-D-19-00092>

Salles, M. M., de Cássia Oliveira, V., Macedo, A. P., Silva-Lovato, C., & Paranhos, H. (2021). Effectiveness of brushing associated with oral irrigation in maintenance of peri-implant tissues and overdentures: Clinical parameters and patient satisfaction. *The Journal of Oral Implantology, 47*(2), 117–123. <https://doi.org/10.1563/aaid-joi-D-19-00092>

Salles, M. M., Oliveira, V. C., Macedo, A. P., do Nascimento, C., Silva‑Lovato, C. H., & Paranhos, H. F. (2021). Brushing associated with oral irrigation in maintaining implants and overdentures hygiene – A randomized clinical trial. *Odontology, 109*(1), 284–294. <https://doi.org/10.1007/s10266-020-00543-7>

Sälzer, S., Slot, D. E., Van der Weijden, F. A., & Dörfer, C. E. (2015). Efficacy of inter-dental mechanical plaque control in managing gingivitis – A meta-review. *Journal of Clinical Periodontology, 42*(16), S92–S105. <https://doi.org/10.1111/jcpe.12363>

Sanders, P. C., Linden, G. J., & Newman, H. N. (1986). The effects of a simplified mechanical oral hygiene regime plus supragingival irrigation with chlorhexidine or metronidazole on subgingival plaque. *Journal of Clinical Periodontology, 13*(3), 237–242. <https://doi.org/10.1111/j.1600-051x.1986.tb01466.x>

Sandra, V. K., Nagireddy, R. R., Nooney, A., & Nadhamuni, M. (2019). Comparison of efficiency of ozone and chlorhexidine subgingival irrigation in orthodontic patients for controlling gingival inflammation. *South African Dental Journal, 74*(2), 82–86. <https://doi.org/10.17159/2519-0105/2019/v74no2a5>

Sandra, V. K., Shaik, Z., Simhadri, G., Obili Govindu Gari, L. R., Siddamreddy, B. P., & Makem, N. (2021). Comparison of laser therapy and ozonated water on gingival inflammation in orthodontic patients with fixed appliances. *Journal of Clinical and Translational Research, 7*(5), 625–630.

Sarlati, F., Azizi, A., Zokaei, N., Mohaghegh, D. N., Hasanzadegan, R. M., & Moradi, H. A. (2016). Comparison of the plaque removal efficacy of Aquajet water flosser and dental floss in adults after a single use (a preliminary study). *Journal of Research in Dental and Maxillofacial Sciences, 1*(4), 16–25.

Sasikumar, P. K., Shanmugam, S., Devi, S. S., & Kirthika, M. (2016). Comparative evaluation of oral irrigator and dental floss as an adjunct to tooth brushing on reduction of plaque and gingivitis – A randomized, single blind clinical study of rural patients. *Journal of Applied Dental and Medical Sciences, 2*(2), 51–61.

Sawan, N., Ben Gassem, A., Alkhayyal, F., Albakri, A., Al-Muhareb, N., & Alsagob, E. (2022). Effectiveness of super floss and water flosser in plaque removal for patients undergoing orthodontic treatment: A randomized controlled trial. *International Journal of Dentistry*, Article 1344258. <https://doi.org/10.1155/2022/1344258>

Schumacher, H. A. (1978). Experiences with the Water-Pik irrigating device in multi-band treatment. *Quintessence International, Dental Digest, 9*(1), 47–49.

Seliger, W. G. (1969). A technique for measuring the penetration of pulsating-jet oral irrigators. *Archives of Oral Biology, 14*(4), 435–436. <https://doi.org/10.1016/0003-9969(69)90100-9>

Selting, W. J., & Bhaskar, S. N. (1973). Target tissue pressures in three water jet devices. *Journal of Dental Research, 52*(3), 441–446. <https://doi.org/10.1177/00220345730520030901>

Selting, W. J., Bhaskar, S. N., & Mueller, R. P. (1972). Water jet direction and periodontal pocket debridement. *Journal of Periodontology, 43*(9), 569–572. <https://doi.org/10.1902/jop.1972.43.9.569>

Sgarbanti, C., Kelekis-Cholakis, A., Stavropoulou, C., & Halas, G. (2021). *A comparison of the efficacy of interdental floss to water flosser around dental implants*. Cochrane Library. <https://www.cochranelibrary.com/central/doi/10.1002/central/CN-01968624/full>

Shalan, L. A., & Al-huwaizi, H. F. (2017). Cleaning efficiency of root canal after irrigation with new irrigation technique: A scanning electron microscopic study. *Iranian Endodontic Journal, 13*(1), 102–107. <https://doi.org/10.22037/iej.v1i1.17285>

Shalan, L. A., Al-huwaizi, H. F., & Fatalla, A. A. (2018). Apical extrusion of debris and irrigants after using different irrigation needles and systems with different depth of penetration: (A comparative study). *Biomedical & Pharmacology Journal, 11*(1), 519–523. <https://doi.org/10.13005/bpj/1402>

Shalan, L. A., Al-huwaizi, H. F., Fatalla, A. A., & Hameed, A. S. (2018). Intra-canal pressure produced by three irrigation system: A comparative study. *Journal of Research in Medical and Dental Science, 6*(5), 161–164.

Sharma, N. C., Lyle, D. M., Qaqish, J. G., Galustians, J., & Schuller, R. (2008). Effect of a dental water jet with orthodontic tip on plaque and bleeding in adolescent patients with fixed orthodontic appliances. *American Journal of Orthodontics & Dentofacial Orthopedics, 133*(4), 565–571. <https://doi.org/10.1016/j.ajodo.2007.12.008>

Sharma, N. C., Lyle, D. M., Qaqish, J. G., & Schuller, R. (2012a). Comparison of two power interdental cleaning devices on plaque removal. *The Journal of Clinical Dentistry, 23*(1), 17–21.

Sharma, N. C., Lyle, D. M., Qaqish, J. G., & Schuller, R. (2012b). Comparison of two power interdental cleaning devices on the reduction of gingivitis. *The Journal of Clinical Dentistry, 23*(1), 22–26.

Silver, J. G., & Chesko, E. E. (1975). The prevalence of periodontal disease in a selected dental population. *Journal of Periodontology, 46*(12), 734–741. <https://doi.org/10.1902/jop.1975.46.12.734>

Slot, D. E., Lyle, D. M., Van der Sluijs, E., Hennequin-Hoenderdos, N., & Van der Weijden, F. (2018). Water flosser compared to interdental brush on bleeding scores and gingival abrasion [Abstract]. *Journal of Dental Research, 97*(Special issue B).

Slot, D. E., Valkenburg, C., & Van der Weijden, G. A. (2020). Mechanical plaque removal of periodontal maintenance patients: A systematic review and network meta-analysis. *Journal of Clinical Periodontology, 47*(Suppl 22), 107–124. <https://doi.org/10.1111/jcpe.13275>

Soltanianzadeh, M. (2020). *Evaluation of complementary supragingival irrigation on the periodontal health in the management of jaw and dentoalveolar fractures using arch bar and dentoalveolar wiring* [Postgraduate Degree in Oral and Maxillofacial Surgery, Kerman University of Medical Sciences]. The WHO Clinical Trials Registry Platform. <https://trialsearch.who.int/>

Soni, S., Sidhu, M. S., & Grover, S. (2012). Comparative evaluation of orthodontic tooth brush and water jet device in the reduction of dental plaque and gingival bleeding in patients with fixed orthodontic appliances. *Journal of Pierre Fauchard Academy (India Section), 26*(4), 147–151. <https://doi.org/10.1016/S0970-2199(12)64001-6>

Southard, G. L., Parsons, L. G., Thomas, L. G., Woodall, I. R., & Jones, B. J. B. (1987). Effect of sanguinaria extract on development of plaque and gingivitis when supragingivally delivered as a manual rinse or under pressure in an oral irrigator. *Journal of Clinical Periodontology, 14*(7), 377–380. <https://doi.org/10.1111/j.1600-051X.1987.tb01539.x>

Stabholz, A., Kettering, J., Aprecio, R., Zimmerman, G., Baker, P. J., & Wikesjö, U. M. (1993). Retention of antimicrobial activity by human root surfaces after in situ subgingival irrigation with tetracycline HCl or chlorhexidine. *Journal of Periodontology, 64*(2), 137–141. <https://doi.org/10.1902/jop.1993.64.2.137>

Stabholz, A., Nicholas, A. A., Zimmerman, G. J., & Wikesjö, U. M. (1998). Clinical and antimicrobial effects of a single episode of subgingival irrigation with tetracycline HCI or chlorhexidine in deep periodontal pockets. *Journal of Clinical Periodontology, 25*(10), 794–800. <https://doi.org/10.1111/j.1600-051x.1998.tb02372.x>

Stelmakh, V., Slot, D. E., & Weijden, G. A. (2017). Self-reported periodontal conditions among Dutch women during pregnancy. *International Journal of Dental Hygiene, 15*(4), e9–e15. <https://doi.org/10.1111/idh.12210>

Sun, J., Tong, D., Sun, C., Wang, X., Zuo, Z., Liu, Y., Qi, L., Kong, L., Luan, X., & Meng, J. (2023). Knowledge, attitude, and practice toward self‑control of dental plaque among patients with periodontal diseases: A cross‑sectional study. *BMC Oral Health, 23*(1), Article 628. <https://doi.org/10.1186/s12903-023-03352-w>

Tamimi, H. A., Thomassen, P. R., & Moser Jr., E. H. (1969). Bacteraemia study using a water irrigation device. *Journal of Periodontology, 40*(7), 4–6.

Tanaka, M., Homma, T., Yamamoto, A., Hashimoto, Y., & Okuda, K. (1968). Microbiological investigation on the effect of oral prophylaxis by Water Pik. Preliminary report. *The Bulletin of Tokyo Dental College, 9*(4), 160–167.

Tardibuono, A. (2021). *Streptococcus mutans differential retention on dental-related substrata* [Master of Science, The State University of New York]. State University of New York at Buffalo ProQuest Dissertations Publishing. <https://www.proquest.com/openview/3fb1270363c88f651a41bc50a12f7a2f/1.pdf?pq-origsite=gscholar&cbl=18750&diss=y>

Tatuskar, P. V., Karmakar, S., Walavalkar, N. N., & Vandana, K. L. (2023). Effect of ozone irrigation and powered toothbrushing on dental plaque, gingival inflammation, and microbial status in institutionalized mentally challenged individuals: A double‑blinded, randomized, controlled clinical trial. *Journal of Indian Society of Periodontology, 27*(3), 315–319. <https://doi.org/10.4103/jisp.jisp_69_22>

Tawakoli, P. N., Sauer, B., Becker, K., Buchalla, W., & Attin, T. (2015). Interproximal biofilm removal by intervallic use of a sonic toothbrush compared to an oral irrigation system. *BMC Oral Health, 15*, Article 91. <https://doi.org/10.1186/s12903-015-0079-6>

Tecco, S., Nota, A., D’Amicantonio, T., Pittari, L., Monti, M., & Polizzi, E. (2022). Effects of an ozonated water irrigator on the plaque index and bleeding index of pregnant women. *Journal of Clinical Medicine, 11*(14), Article 4107. <https://doi.org/10.3390/jcm11144107>

Tempel, T. R., Marcil, J. F., & Seibert, J. S. (1975). Comparison of water irrigation and oral rinsing on clearance of soluble and particulate materials from the oral cavity. *Journal of Periodontology, 46*(7), 391–396. <https://doi.org/10.1902/jop.1975.46.7.391>

Terry, M. A., Narayana, N., & Kaldahl, W. B. (2014). Gingival foreign body granuloma in conjunction with the use of a dental water jet: A case report. *General Dentistry, 62*(5), 37–39.

Toto, P. D., Evans, C. L., & Sawinski, V. J. (1969). Effects of water jet rinse and toothbrushing on oral hygiene. *Journal of Periodontology, 40*(5), 296–298. <https://doi.org/10.1902/jop.1969.40.5.296>

Tütüncüoğlu, S., Cetinkaya, B. O., Pamuk, F., Avci, B., Keles, G. C., Kurt-Bayrakdar, S., & Lütfioğlu, M. (2022). Clinical and biochemical evaluation of oral irrigation in patients with peri-implant mucositis: A randomized clinical trial. *Clinical Oral Investigations, 26*(1), 659–671. <https://doi.org/10.1007/s00784-021-04044-x>

Tyler, D., Kang, J., & Goh, H. H. (2023). Effectiveness of Waterpik® for oral hygiene maintenance in orthodontic fixed appliance patients: A randomised controlled trial. *Journal of Orthodontics, 50*(4), 367–377. <https://doi.org/10.1177/14653125231173708>

Van Dijk, L. J., Lie, M. A., Van den Heuvel, E. R., & Van der Weijden, G. A. (2018). Adult periodontitis treated with a new device for subgingival lavage—a randomized controlled clinical trial using a split-mouth design. *International Journal of Dental Hygiene, 16*(4), 559–568. <https://doi.org/10.1111/idh.12344>

Varela-Centelles, P., Bugarín-González, R., Blanco-Hortas, A., Estany-Gestal, A., Varela-Centelles, A., & Seoane-Romero, J. M. (2020). Family nurses, oral hygiene, and educational implications: A cross-sectional study. *Stomatologija, 22*(4), 120–124.

Vignarajah, S., Newman, H. N., & Bulman, J. (1989). Pulsated jet subgingival irrigation with 0.1% chlorhexidine, simplified oral hygiene and chronic periodontitis. *Journal of Clinical Periodontology, 16*(6), 365–370. <https://doi.org/10.1111/j.1600-051x.1989.tb00006.x>

Volman, E. I., Stellrecht, E., & Scannapieco, F. A. (2021). Proven primary prevention strategies for plaque-induced periodontal disease – An umbrella review. *Journal of the International Academy of Periodontology, 23*(4), 350–367.

Waki, M. Y., Jolkovsky, D. L., Otomo-Corgel, J., Lofthus, J. E., Nachnani, S., Newman, M. G., & Flemmig, T. F. (1990). Effects of subgingival irrigation on bacteremia following scaling and root planing. *Journal of Periodontology, 61*(7), 405–411. <https://doi.org/10.1902/jop.1990.61.7.405>

Walsh, M., Heckman, B., Leggott, P., Armitage, G., & Robertson, P. B. (1989). Comparison of manual and power toothbrushing, with and without adjunctive oral irrigation, for controlling plaque and gingivitis. *Journal of Clinical Periodontology, 16*(7), 419–427. <https://doi.org/10.1111/j.1600-051x.1989.tb01670.x>

Walsh, T. F., Glenwright, H. D., & Hull, P. S. (1992). Clinical effects of pulsed oral irrigation with 0.2% chlorohexidine digluconate in patients with adult periodontitis. *Journal of Clinical Periodontology, 19*(4), 245–248. <https://doi.org/10.1111/j.1600-051x.1992.tb00461.x>

Walsh, T. F., Unsal, E., Davis, L. G., & Yilmaz, O. (1995). The effect of irrigation with chlorhexidine or saline on plaque vitality. *Journal of Clinical Periodontology, 22*(3), 262–264. <https://doi.org/10.1111/j.1600-051x.1995.tb00144.x>

Wang, Y., Gao, H., Chang, L., Xu, J., Zhou, X., Zhang, C., & Peng, Q. (2023). Efficient removal of dental plaque biofilm from training typodont teeth via water flosser. *Bioengineering, 10*(9), Article 1061. <https://doi.org/10.3390/bioengineering10091061>

Watt, D. L., Rosenfelder, C., & Sutton, C. D. (1993). The effect of oral irrigation with a magnetic water treatment device on plaque and calculus. *Journal of Clinical Periodontology, 20*(5), 314–317. <https://doi.org/10.1111/j.1600-051x.1993.tb00366.x>.

Watts, E. A., & Newman, H. N. (1986). Clinical effects on chronic periodontitis of a simplified system of oral hygiene including subgingival pulsated jet irrigation with chlorhexidine. *Journal of Clinical Periodontology, 13*(7), 666–670. <https://doi.org/10.1111/j.1600-051x.1986.tb00863.x>

Wheatcroft, M. G., & Sciantarelli, E. (1974). The effect of oral water irrigation on the prevention of gingival inflammation. *The Journal of the American Society for Preventive Dentistry, 4*(4), 38–39.

Wikesjö, U. M., Reynolds, H. S., Christersson, L. A., Zambon, J. J., & Genco, R. J. (1989). Effects of subgingival irrigation on *A. actinomycetemcomitans*. *Journal of Clinical Periodontology, 16*(2), 116–119. <https://doi.org/10.1111/j.1600-051x.1989.tb01623.x>

Winter, A. A. (1982). Rapid destruction caused by a water-irrigating device. *Dental Health, 21*(5), 18–20.

Wolff, L. F., Bakdash, M. B., Pilhlstrom, B. L., Bandt, C. L., & Aeppli, D. M. (1989). The effect of professional and home subgingival irrigation with antimicrobial agents on gingivitis and early periodontitis. *Journal of Dental Hygiene, 63*(5), 222–225, 241.

Worthington, H. V., MacDonald, L., T., P. P., Sambunjak, D., Johnson, T. M., Imai, P., & Clarkson, J. E. (2019). Home use of interdental cleaning devices, in addition to toothbrushing, for preventing and controlling periodontal diseases and dental caries. *Cochrane Database of Systematic Reviews*. <https://doi.org/10.1002/14651858.CD012018.pub2>

Wright, W. E., & Tempel, T. R. (1974). Effect of oral hygiene on clearance of soluble chemotactic bacterial products from human saliva. *Journal of Periodontology, 45*(3), 134–138. <https://doi.org/10.1902/jop.1974.45.3.134>

Xu, X., Zhou, Y., Liu, C., Zhao, L., Zhang, L., Li, H., Li, Y., & Cheng, X. (2023). Effects of water flossing on gingival inflammation and supragingival plaque microbiota: A 12‑week randomized controlled trial. *Clinical Oral Investigations, 27*(8), 4567–4577. <https://doi.org/10.1007/s00784-023-05081-4>

Yamada, J., Peng, K., Kokubun, T., Takiguchi, T., Li, G., Yamamoto, M., & Soyama, H. (2018). Optimization of a cavitating jet for removing dental plaque from the surface of the screw of an implant and its practical application. *Journal of Biomechanical Science and Engineering, 13*(2). <https://doi.org/10.1299/jbse.17-00102>

Yamada, J., Takiguchi, T., Saito, A., Odanaka, H., Soyama, H., & Yamamoto, M. (2017). Removal of oral biofilm on an implant fixture by a cavitating jet. *Implant Dentistry, 26*(6), 904–910. <https://doi.org/10.1097/ID.0000000000000681>

Yeo, H. J., Kim, K. M., & Kim, H. J. (2021). The change in oral microorganisms in orthodontic patients depending on the use of Waterpiks. *Natural Volatiles and Essential Oils, 8*(5), 2015–2023.

York, T. A., & Dunkin, R. T. (1967). Control of periodontal problems in orthodontics by use of water irrigation. *American Journal of Orthodontics, 53*(9), 639–650. <https://doi.org/10.1016/0002-9416(67)90175-3>

Yuen, H. K. (2013). Effect of a home telecare program on oral health among adults with tetraplegia: A pilot study. *Spinal Cord, 51*(6), 477–481. <https://doi.org/10.1038/sc.2012.176>

Yuen, H. K., & Pope, C. (2009). Oral home telecare for adults with tetraplegia: A feasibility study. *Special Care in Dentistry, 29*(5), 204–209. <https://doi.org/10.1111/j.1754-4505.2009.00094.x>

Zellmer, I. H., Couch, E. T., Berens, L., & Curtis, D. A. (2020). Dental Hygienists’ knowledge regarding dental implant maintenance care: A national survey. *Journal of Dental Hygiene, 94*(6), 6–15.

Zhao, R., Liu, S., Liu, Y., & Cui, S. (2022). Adjunctive use of active compounds such as chlorhexidine in the nonsurgical treatment of peri-implant mucositis for oral health: A systematic review and meta-analysis. *Oxidative Medicine and Cellular Longevity, 2022*, Article 2312784. <https://doi.org/10.1155/2022/2312784>
